# Translation Reinitiation Relies on the Interaction between eIF3a/TIF32 and Progressively Folded *cis*-Acting mRNA Elements Preceding Short uORFs

**DOI:** 10.1371/journal.pgen.1002137

**Published:** 2011-07-07

**Authors:** Vanda Munzarová, Josef Pánek, Stanislava Gunišová, István Dányi, Béla Szamecz, Leoš Shivaya Valášek

**Affiliations:** 1 Laboratory of Regulation of Gene Expression, Institute of Microbiology, Academy of Sciences of the Czech Republic, Prague, Czech Republic; 2 Laboratory of Bioinformatics, Institute of Microbiology, Academy of Sciences of the Czech Republic, Prague, Czech Republic; Thomas Jefferson University, United States of America

## Abstract

Reinitiation is a gene-specific translational control mechanism characterized by the ability of some short upstream uORFs to retain post-termination 40S subunits on mRNA. Its efficiency depends on surrounding *cis*-acting sequences, uORF elongation rates, various initiation factors, and the intercistronic distance. To unravel effects of *cis*-acting sequences, we investigated previously unconsidered structural properties of one such a *cis*-enhancer in the mRNA leader of *GCN4* using yeast genetics and biochemistry. This leader contains four uORFs but only uORF1, flanked by two transferrable 5′ and 3′ *cis*-acting sequences, and allows efficient reinitiation. Recently we showed that the 5′ *cis*-acting sequences stimulate reinitiation by interacting with the N-terminal domain (NTD) of the eIF3a/TIF32 subunit of the initiation factor eIF3 to stabilize post-termination 40S subunits on uORF1 to resume scanning downstream. Here we identify four discernible reinitiation-promoting elements (RPEs) within the 5′ sequences making up the 5′ enhancer. Genetic epistasis experiments revealed that two of these RPEs operate in the eIF3a/TIF32-dependent manner. Likewise, two separate regions in the eIF3a/TIF32-NTD were identified that stimulate reinitiation in concert with the 5′ enhancer. Computational modeling supported by experimental data suggests that, in order to act, the 5′ enhancer must progressively fold into a specific secondary structure while the ribosome scans through it prior uORF1 translation. Finally, we demonstrate that the 5′ enhancer's stimulatory activity is strictly dependent on and thus follows the 3′ enhancer's activity. These findings allow us to propose for the first time a model of events required for efficient post-termination resumption of scanning. Strikingly, structurally similar RPE was predicted and identified also in the 5′ leader of reinitiation-permissive uORF of yeast *YAP1*. The fact that it likewise operates in the eIF3a/TIF32-dependent manner strongly suggests that at least in yeasts the underlying mechanism of reinitiation on short uORFs is conserved.

## Author Summary

Protein synthesis is a fundamental mechanism capturing the rejuvenation of DNA–encoded genetic information by its translation into molecular effectors—proteins. Its regulation can be used to change the protein content and thus to adapt a cell to changing environmental conditions. Translation requires mRNAs delivering genetic information of corresponding genes, tRNAs carrying amino-acids, ribosomes as the molecular translators, and accessory proteins/factors facilitating the entire process. There are numerous regulatory mechanisms that modulate translation at its various stages. Here we describe one such a translational control mechanism called reinitiation. Most eukaryotic mRNAs contain only a single translatable gene (ORF); however, in many of them this gene is preceded by a short coding sequence (uORF) that is in some cases translated first. In order to reinitiate translation on the downstream main ORF, a ribosome has to stay bound to mRNA after it has terminated short uORF translation. This requires a concerted action of specific mRNA elements surrounding the uORF and selected initiation factors. Our results delineate how these key players interact with each other and suggest a sequence of general events that ought to take place on short uORF to enable the ribosome to reach and translate the main ORF downstream.

## Introduction

Translation of the majority of eukaryotic mRNAs encoding almost exclusively only a single large open reading frame (ORF) is initiated by the canonical mechanism involving formation of the 48S pre-initiation complex (PIC) at the mRNA's 5′ cap structure followed by scanning through the 5′ untranslated region (UTR) for usually the nearest AUG start codon (reviewed in [1]). According to recent reports, however, in approximately 13% of yeast and 50% of human transcripts the main ORF is preceded by one or more short upstream ORFs (uORFs) [1], [2], consisting of the AUG start codon and at least one additional coding triplet.

Presence of a short uORF in mRNA's 5′ UTR generally leads to significant reduction in expression of a main ORF [2], the degree of which depends on the “strength” of the nucleotide context surrounding the uORF's initiating AUG (called the Kozak consensus sequence) [3]. Short uORFs with a relatively poor initiation context can be skipped by at least some 48S PICs via leaky scanning, which decreases their inhibitory impact. On the other hand, there is growing evidence that there are many non-AUG-initiating short uORFs that, if in a good context, may serve as very potent inhibitors [4], [5]. Short uORFs may also down-regulate expression of a main ORF by their special ability to mediate ribosome stalling at coding or termination codons, or by influencing the mRNA stability through the Nonsense Mediated Decay (NMD) pathway (reviewed in [6]). On the other side of the spectrum of short regulatory uORFs are those that permit the small ribosomal subunit to stay mRNA-bound post-termination and resume scanning for efficient reinitiation (REI) downstream.

It has been shown that the ability of some uORFs to retain the 40S subunit on the same mRNA molecule after it has terminated translation at the uORF's stop codon depends on: (i) *cis*–acting mRNA features, (ii) the time required for the uORF translation, which is determined by the relative length of a short uORF and the translation elongation rates, and (iii) on various initiation factors (for review see [6]–[8]). The last two requirements are united in the idea that eIFs important for promoting reinitiation remain at least transiently associated with the elongating ribosome, and that increasing the uORF length or the ribosome transit time increases the likelihood that these factors are dropped off [9]. There is now genetic evidence for this hypothesis showing that in yeast *S. cerevisiae* eIF3 remains 80S-bound for several rounds of elongation and critically enhances the REI capacity of post-termination 40S ribosomes [10] (see also below). With respect to *cis-*acting features, with the exception of the uORF-mediated translational control of the budding yeast *GCN4* described below, there is virtually nothing known about what other REI-promoting mRNA features are required. Finally, REI efficiency is also directly dependent on (iv) the distance between the uORF termination codon and a downstream initiation codon owing to the fact that the rescanning PICs require a certain time for *de novo* recruitment of the eIF2•GTP•Met-tRNA_i_^Met^ ternary complex (TC) to be able to decode the next AUG start site [11].

**Figure 1:**
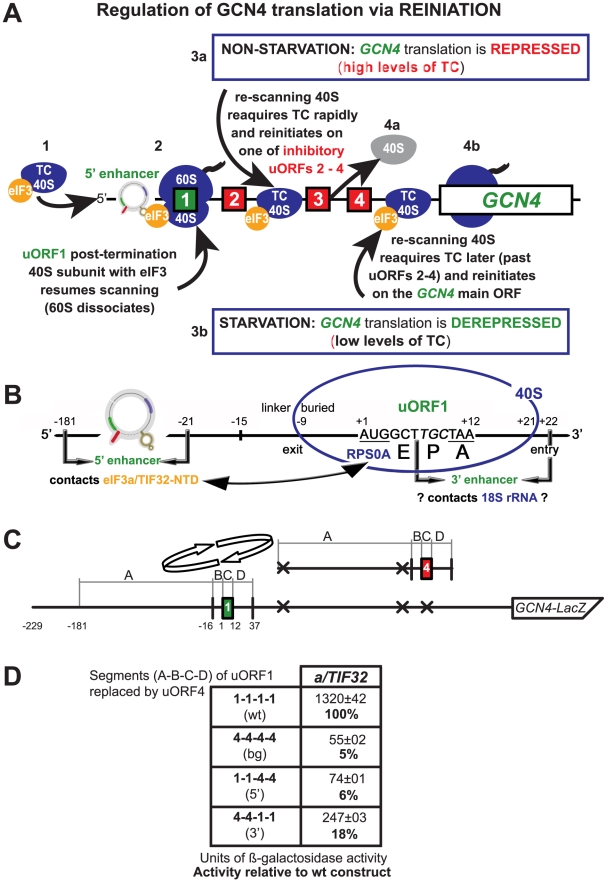
REI-promoting activity of the 5′ sequences of uORF is strictly dependent on that of the 3′ enhancer. (A) Schematic of the *GCN4* mRNA leader showing distribution of all four short uORFs (REI-permissive uORF1 is labeled green; REI-non-permissive uORFs 2–4 are labeled red), the predicted structure of the uORF1's 5′ *cis*-acting sequences (5′ enhancer) defined in this study, 40S- and 80S-bound eIF3, and the description of the mechanism of the *GCN4* translation control. The 3a and 4a “*GCN4*-expression repressed” steps take places under non-starvation conditions with abundant ternary complex (TC) levels, whereas the 3b and 4b “*GCN4*-expression derepressed” steps occur under starvation condition with limited supply of the TC (see text for further details). (B) Schematic showing predicted position of the 40S ribosome terminating at the stop codon of uORF1 from the *GCN4* mRNA leader (adapted from [10]). E, P, and A sites of the 40S ribosome are aligned with the last two coding triplets and the TAA stop codon; entry and exit pores of the mRNA binding channel are labeled. The locations of the uORF1's 5′ sequences/enhancer (interacting with the NTD of a/TIF32), the 3′ enhancer (proposed to contact 18S rRNA), linker, and buried parts of the sequences upstream of uORF1 are indicated. The interaction between the a/TIF32-NTD and the small ribosomal protein RPS0A is depicted by a double headed arrow. (C) Schematic showing the *GCN4-lacZ* construct containing solitary uORF1, the surrounding sequences of which were divided into four separate segments (A1–D1; see text for further details). Arrows indicate replacements of these segments with the corresponding segments (A4–D4) surrounding uORF4, shown to the right of the arrows. (D) Various *GCN4-lacZ* constructs with the segment's combinations indicated in the first column were introduced into the YBS47 strain. The resulting transformants were pre-cultured in minimal media overnight, diluted to OD_600_ ∼0.35, grown for additional 6 hrs and the β-galactosidase activities were measured in the WCEs and expressed in units of nmol of o-nitrophenyl-b-D-galactopyranoside hydrolyzed per min per mg of protein. The mean values and standard deviations obtained from at least 3 independent measurements with three independent transformants, and activity in the mutant constructs relative to wt, respectively, are given in right column.

The *GCN4* mRNA encodes a transcriptional activator of mainly amino acid biosynthetic genes and its leader sequence contains four short uORFs (Figure 1A). Independent of amino acid availability, most ribosomes translate the first REI-permissive uORF (uORF1) and, following termination, about a half of them resumes scanning downstream. When amino acid levels are high, re-scanning ribosomes reacquire the TC relatively rapidly afterward and preferentially reinitiate at one of the last three uORFs, none of which supports efficient REI (see our model in Figure 1A). When amino acid levels are low, deacylated tRNAs accumulate, activating the eIF2α kinase GCN2. As a result, the TC levels are decreased and the re-scanning ribosomes must travel for a longer period till they have rebound the TC. This significantly increases the likelihood of bypassing all three REI-nonpermissive uORFs to reach the *GCN4*′s start codon. Thus, whereas the global protein synthesis is significantly down-regulated, translational expression of *GCN4* is concurrently induced (derepressed). A failure to derepress *GCN4* expression is called the Gcn^-^ (*g*eneral *c*ontrol *n*onderepressible) phenotype. A similar regulatory mechanism has been also shown to govern expression of for example the mammalian functional homologue of *GCN4*, the *ATF4* transcription factor [12].

The pressing question of why ribosomes readily reinitiate after translation of uORF1 but not the other uORFs has baffled the translational field for many years. Mutational analyses indicated that AU-rich sequences surrounding the stop codon of uORF1 (dubbed the 3′ enhancer herein) might favor resumption of scanning and REI [13] (Figure 1B). In addition, sequences 5′ of uORF1 were also shown to be critical for efficient REI [14] (Figure 1B). In contrast to the 3′ enhancer, the molecular mechanism of which remains to be elucidated, the molecular contribution of the 5′ sequences has been recently proposed on the basis of our characterization of the N-terminal truncation of the a/TIF32 subunit of eIF3 [10]. The N-terminal domain (NTD) of a/TIF32 was previously shown to interact with the small ribosomal protein RPS0A *in vitro* [15], and we subsequently found that the N-terminal truncation in *a/tif32-Δ8* severely reduced association of eIF3 and its associated eIFs with the small ribosomal subunit *in vivo* [10]. (RPS0A is positioned near the mRNA exit pore on the solvent side of the 40S subunit [16]). Unexpectedly, however, *a/tif32-Δ8* also produced a severe Gcn^-^ phenotype as it failed to up-regulate *GCN4* expression under starvation conditions by preventing the post-termination ribosomes from resuming scanning downstream of the uORF1's stop codon. Detailed genetic analysis suggested that besides RPS0A, the a/TIF32-NTD also interacts with a yet to be identified element(s) within the uORF1's 5′ sequences. Together our findings led to a working model in which wild-type eIF3 remains at least transiently associated with the translating 80S ribosome, and if it does not drop off prior to termination, the a/TIF32-NTD interacts with the 5′ sequences to permit ribosomal recycling of only the large 60S subunit while aiding to preserve the small subunit on the *GCN4* mRNA [10] (Figure 1A and 1B). This last step serves as a critical prerequisite for subsequent resumption of scanning by the 40S subunit for REI downstream. Interestingly, we have only recently showed that the eIF3g/TIF35 subunit of yeast eIF3 also critically contributes to this process, but the mechanism of its involvement seems to differ from that of a/TIF32 [17]. Besides the uORF1 of *GCN4*, there is another well described example of a REI-permissive uORF in yeast represented by uORF of the *YAP1* gene, an AP1-like transcription factor [18]. The intriguing question is whether the molecular aspects of its reinitiation mechanism are similar to that of *GCN4*'s uORF1, which could indicate a broad mechanistic conservation of reinitiation on short uORFs.

In this study we have subjected the ∼220-nt long 5′ sequences of uORF1 as well as the first 200 amino acid residues of the a/TIF32-NTD to an in-depth mutational analysis to identify specific elements/regions required for their common REI-promoting activity. Four such elements designated REI-promoting elements (RPEs) are described that together make up what we now call the 5′ enhancer. In addition, two distal regions within the NTD of a/TIF32 were identified and shown to promote REI in the 5′ enhancer-dependent manner. Enhanced computer modeling taking into account a progressive character of mRNA folding combined with classical enzymatic probing surprisingly revealed that the 5′ enhancer contains only two well-defined structural features in a 9-nt long stem and a double-circle hairpin representing the RPEs ii. and iv., respectively. Strikingly, a similar structural motif working in concert with the a/TIF32-NTD was also found upstream of the REI-permissive uORF of *YAP1*. These findings thus strongly suggest existence of a conserved short uORF-mediated mechanism of reinitiation, whereby the a/TIF32-NTD of the post-termination 80S-bound eIF3 must contact the specifically folded *cis*-acting REI-promoting elements 5′ of uORF in order to facilitate efficient resumption of scanning of the 40S ribosomal subunit.

## Results

### The 5′ and 3′ sequences of uORF1 closely cooperate in stimulating efficient REI

A considerable difference in efficiency of resumption of scanning following translation of uORF1 versus uORF4 in the *GCN4* mRNA leader is thought to be attributable to the distinct sequences surrounding the termination codons of these two uORFs. Replacing the last codon and 10 nt downstream of the uORF1 stop codon (Figure 1B, dubbed the 3′ enhancer) with the corresponding nucleotides from uORF4 was sufficient to make uORF1 as inhibitory for REI on *GCN4* as is uORF4 [13]. Similarly, sequences located in the leader region >20 nt upstream of the AUG start codon of uORF1 (Figure 1B) were also shown to be critically required for efficient REI downstream [14]. However, individual contributions of both of these stimulatory sequences to the overall REI efficiency have never been directly compared in a single experiment. To do that, we divided the two uORFs and their surrounding sequences into four segments: segment A (166 bp in length from position -181 to -16 relative to the AUG start codon corresponding to the 5′ REI-promoting sequences of uORF1); segment B (15-bp long segment (−15 to −1) designated previously as linker [10]); segment C (3 coding triplets and a termination codon); and segment D (25 bp downstream from the uORF stop codon including the aforementioned 3′ enhancer of uORF1) (Figure 1C). It should be noted that the A segment of uORF4 has the start codons of the preceding uORFs 2 and 3 mutated out to compare the effects of only uORFs 1 and 4. Also, in contrast to A, C and D segments, the sequence corresponding to the B-linker region of uORF1 was previously shown to play a negligible role for efficient REI [19]. Three hybrid uORFs were constructed by the substitution of some or all of uORF1 segments with the corresponding segments derived from uORF4 in the *GCN4-lacZ* construct lacking all three uORFs naturally occurring downstream of uORF1 (compare Figure 1A and 1C). When all four uORF1 segments were replaced by the corresponding uORF4 segments (Figure 1D; row 2 (construct 4-4-4-4)), the *GCN4-lacZ* expression dropped by ∼20-fold to the background level (Figure 1D, row 2 [bg] versus 1 [wt]) in accord with previous findings demonstrating the two uORFs' highly disparate capacities to promote efficient REI [19]. Selective replacements of either the 5′ sequences or the entire 3′ enhancer (row 4 (construct 4-4-1-1) versus row 3 (1-1-4-4)) of uORF1 resulted in 6-fold or 17-fold reductions in β-galactosidase activities, respectively. These data indicate that both elements closely co-operate to promote highly effective REI downstream of uORF1, but probably by mechanistically distinct processes. Interestingly, whereas the 3′ enhancer is sufficient to stimulate resumption of scanning to at least some degree (by ∼13% after background subtraction), the 5′ sequences are not. This fact could imply that the 3′ enhancer acts first and its stimulatory activity is required for the subsequent action of the 5′ enhancing sequences. It is important to note that the transfer of both sequence elements into the sequence context of REI-nonpermissive uORF4 converts it into a REI-permissive uORF [19]. Hence the mechanism of their combined action appears to be general, not specific to uORF1 only.

### The 5′ sequences of uORF1 contain at least three REI-promoting elements one of which operates in an a/TIF32-NTD–dependent manner

Whereas the molecular mechanism by which the 3′ enhancer promotes REI is unknown, our recent genetic epistasis analysis suggested that the 5′ sequences (in segment A) emerging from the 40S mRNA exit channel promote REI by interacting with the NTD of a/TIF32 upon termination on the uORF1 stop codon. This interaction was proposed to stabilize association of the post-termination 40S subunit with the *GCN4* mRNA so that it could resume scanning for REI downstream [10]. Partial deletions of the 5′ sequences in the *GCN4-lacZ* construct containing solitary uORF1 had severe deleterious effects on efficiency of REI in the wt *a/TIF32* background but not in the *a/tif32Δ* cells expressing a viable *a/tif32-Δ8* allele lacking sequences encoding the extreme N-terminal 200 amino acid residues. Given that the 5′ enhancing sequences comprise a rather long stretch of ∼160 nt, however, it is fairly unlikely that such a long segment contacts eIF3 bound to the 40S as a whole. In fact, previously published data suggested that it may consist of at least two critical elements, as deletions of 40, 80 and 120 nt from nt −21 upstream reduced the *GCN4-lacZ* expression by a similar fold (from 2.5- to 3-fold), whereas the largest deletion of 160 nt resulted in ∼6-fold reduction [14].

**Figure 2:**
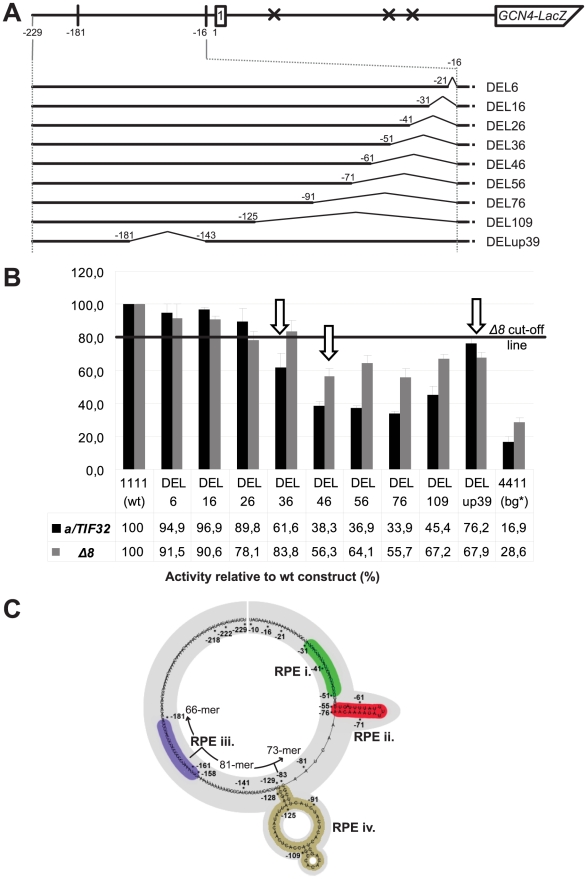
The 5′ sequences of uORF1 contain at least three REI-promoting elements (RPEs), one of which operates in the a/TIF32-NTD–dependent manner. (A) Schematic showing the solitary uORF1 *GCN4-lacZ* construct with the battery of deletions in the uORF1's 5′ UTR defined below and used in panel B. (B) The YBS47 (*a/TIF32*) and YBS53 (*a/tif32-Δ8*) strains were introduced with the *GCN4-lacZ* deletion constructs described in panel A and Figure 1D and analyzed as in Figure 1D, except that YBS53 was grown for 8 hours. Arrows indicate constructs defining the individual RPEs; please see corresponding text for the definition of the *Δ8* cut-off line. (C) *In silico* prediction of the secondary structure of the 5′ enhancer of uORF1 (nt −229 through -10) carried out with the RNA fold software [21]. Four individual RPEs identified in panel B and Figure 4 are labeled and color-coded. Division into three segments used for computer modeling is indicated.

In order to precisely map the minimal region(s) responsible for the REI-promoting role of the uORF1's 5′ sequences that work in concert with the a/TIF32-NTD, the 5′ sequences were progressively deleted (beginning at a position −16 nt relative to the uORF1 AUG codon) in a *GCN4-lacZ* construct containing solitary uORF1 (Figure 2A). For example, constructs DEL6 and DEL36 had internal deletions of 6 nt (from −16 to −21) and 36 nt (from −16 to −51), respectively. As a specific background control, the 4-4-1-1 construct devoid of the entire 5′ enhancing sequences (defined in Figure 1C) was routinely used (bg*). All deletion constructs were expressed in both the *a/TIF32* wt and *a/tif32-Δ8* mutant strains and the levels of β-galactosidase activities were measured in at least three independent experiments with three individual transformants in triplicates for each construct. These values were then expressed relative to the value obtained with the wt uORF1-*GCN4-lacZ* construct that was set to 100% in both strains. The mean values of the resulting percentages (with standard deviations) from all experiments were calculated and plotted (Figure 2B). We opted for this percentage expression because it enables a better comparison of the effects of the 5′ sequences deletions on relative β-galactosidase activities independently in each strain. It is important to remember, however, that the *a/tif32-Δ8* mutation itself reduces expression of the *GCN4-lacZ* from the uORF1-*GCN4-lacZ* constructs by ∼70% when compared to wt *a/TIF32* [10], and the chosen way of data presentation does not reflect this dramatic difference in activities. Owing to this “scaling up” we set a cut-off line of 80% for changes that are considered significant in the *a/tif32-Δ8* mutant cells. (For comparison, the raw, not-normalized data for some of the constructs are shown in Figure S1A and S1B). It is also important to note that mRNAs produced from all *GCN4-lacZ* constructs used throughout the study are highly stable in both wt and *a/tif32-Δ8* strains thanks to the fact that they all contain an intact stabilizer element (STE) that protects the natural *GCN4* mRNA from NMD [10], [20] (Figure S1C).

As shown in Figure 2B, deletions of up to 16 nt from the 3′ end of the 5′ sequences (DEL6 and DEL16) did not produce any significant changes in the *GCN4-lacZ* expression in the wt cells. In contrast, larger deletions of 26, 36, and mainly of 46 nt (DEL 26, DEL36, and DEL46) reduced β-galactosidase activities by ∼10%, ∼40%, and ∼60%, respectively. None of the largest deletions (DEL56 through DEL109) decreased the levels of *GCN4-lacZ* expression any further (i.e. above 60% of DEL46). In striking contrast to the wt cells, DEL36 had virtually no effect in the *a/tif32-Δ8* cells, whereas DEL46 led to a substantial drop in activity (by ∼40%). None of the largest deletions decreased the *GCN4-*lacZ expression in *a/tif32-Δ8* any further, just like in *a/TIF32*. Taken together, these results indicate the existence of two REI-promoting elements (RPE) falling between nt −31 and −61. The first element (RPE i.; −31 through −51) appears to function in the a/TIF32-NTD-dependent manner, since its removal in DEL36 shows genetic epistasis (non-additive phenotype) with the *a/tif32-Δ8* mutation. The second REI-promoting element (RPE ii.; −51 through at least -61), however, operates independently of the a/TIF32-NTD as its deletion together with the RPE i. in DEL46 produced a sharp decrease in β-galactosidase activities in both wt as well as mutant cells.

Next we wanted to examine whether the far upstream sequence between nt in positions −143 and −181 constitutes yet another REI-promoting element of the 5′ sequences as originally proposed by Grant and co-workers [14]. Towards this end, we deleted the corresponding region from the wt leader in DELup39 (Figure 2A) and observed ∼25% and >30% reductions of activities in wt and *a/tif32-Δ8* mutant cells, respectively (Figure 2B). These results thus unambiguously reveal the presence of a third REI-promoting element (RPE iii.; −143 through −181) in the 5′ sequences of uORF1 that seems to be less potent than the other two and that enhances the efficiency of REI in the a/TIF32-NTD-independent fashion. To conclude, our deletion analysis identified three RPEs that together make up what we designate the 5′ enhancer of uORF1 thereafter.

### *In silico* prediction of the secondary structure of the 5′ enhancer of uORF1

Having identified three RPEs in the 5′ enhancer of uORF1, we wished to predict a potential secondary structure that the entire 220 nt long segment of the uORF1 5′ UTR might progressively fold into during scanning for, translation elongation of, and termination on uORF1. Note that we excluded the most 3′ terminal 9 nt from our analysis as they are highly likely buried in the mRNA binding channel of the 80S ribosome terminating at uORF1 [10]. The computer modeling was carried out by the RNA fold software [21]. Our prediction was based on two facts: 1) the 5′ enhancer is not a standalone molecule with a rigid structure; its fold forms and changes dynamically as the sequence emerges from the ribosomal mRNA exit pore; and 2) the overall underrepresentation of Guanosines (the nucleotide composition of the entire 5′ UTR of uORF1 is: A 40%, C 22%, G 7%, T 31%). Since the Gs are missing especially at the very 5′ end of the sequence, we reasoned that their absence might leave this region unstructured, after it has emerged from the mRNA exit channel, owing to the fact that no local G–C pairs can be formed. To take these assumptions into account in our model, we divided the 5′ UTR of uORF1 into three consecutive segments represented by the extreme 5′ end 66-mer (AU-rich), the middle 81-mer, and the extreme 3′ end 73-mer that is also AU-rich. We first folded the extreme 5′ segment and found that, in agreement with our reasoning, the 66-mer showed no predictions of any secondary structures (Figure 2C). Importantly, it is believed that the AU-rich sequences have a stronger tendency to interact with proteins than those rich in Gs [22]. Hence it is conceivable that the extreme 5′ AU-rich RNA stretch remains unstructured to engage in binding to ribosomal proteins and/or translation factors situated in the vicinity of the mRNA exit pore. Given this potential, we further stipulated that this 66-mer would not directly pair with the downstream sequences gradually leaving the exit channel during ribosomal scanning. To account for this, we added the middle 81-mer to the 66-mer and modeled the folding of the resulting 147-mer by blocking potential contacts between both individual segments. As a result, a short double-circle hairpin relatively GC-rich was predicted to form at the very 3′ end of the 147-mer (Figure 2C). Interestingly, the same hairpin formed when the complete sequence of the 5′ UTR of uORF1 was analyzed by RNA fold without any restraints (data not shown), and, furthermore, when homologues sequences from numerous yeast species were subjected to computer modeling (JP and LV, unpublished observations). These results indicate that the double-circle hairpin is a conserved structure, at least among various yeasts, that may have a functional significance in the translational control mechanism of *GCN4* (see below). Finally, we added the remaining extreme 3′ end segment to the pre-folded 147-mer and sought predictions of the overall structure of the 5′ sequences. As shown in Figure 2C, the 73-mer remained mostly unfolded with the exception of a 9-nt long stem loop, situated only 6 nt downstream of the 3′ end of the double-circle hairpin, with one 3-nt topical bulge and one 1-nt bulge close to its 3′ end. Taken together with our genetic deletion analysis presented above, we propose that both the RPE i. and RPE iii. remain unstructured, whereas the RPE ii. folds into a stable stem loop with two bulges (Figure 2C).

### RNA structure probing of the 5′ enhancer of uORF1

**Figure 3:**
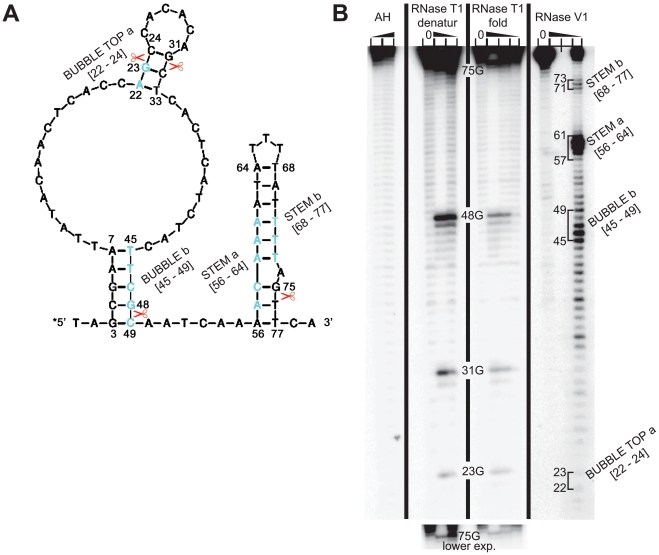
RNA structure probing of the 5′ enhancer of uORF1. (A) *In silico* prediction of the secondary structure of the 79-nt segment of the 5′ enhancer of uORF1 that was subjected to enzymatic probing. Scissors and light blue residues indicate cleavage sites of T1 and V1 RNases shown in panel B, respectively. (B) RNA structure probing of the commercially synthesized uORF1's 5′ enhancer segment comprising the RPEs ii. and iv. The latter 79-mer was 5′-end labeled with [γ-^32^P]-ATP and subjected to limited RNase cleavage using RNases T1 and V1 under denaturing (denatur) or folding-promoting (fold) conditions. Sites of cleavage were identified by comparison with a ladder of bands created by limited alkaline hydrolysis of the RNA (AH) and by the position of known RNase T1 cuts, determined empirically. Predicted double-stranded regions are indicated on the right-hand side of the panel and the shorter exposition of the upper portion of the gel showing T1 cuts is shown at the bottom of the panel.

To test our computer predictions experimentally, we subjected a commercially synthesized 79-mer containing both the double-circle hairpin and the RPE ii. stem to enzymatic probing. (The 79-mer that was chosen based on RNA fold predictions starts 2 nt before the hairpin and ends 2 nt after the RPE ii. stem (Figure 3A).) The 79-mer was 5′-end labelled by T4 polynucleotide kinase with [^32^P]-γATP, heated at 90°C for 3 minutes, slowly cooled down to room temperature to stimulate proper re-folding, and probed by RNases T1 and V1 prior to analysis on denaturing polyacrylamide gels. As shown in Figure 3B, the data for enzymatic probing were in good agreement with the computationally predicted secondary structure of this 5′ enhancer section. Formation of all three stems, two of which occur in the double-circle hairpin [nt 3–7 base-paired with nt 45–49; and nt 22–24 base-paired with nt 31–33], and the third forms the RPE ii [nt 56–64 base-paired with nt 68–77], was confirmed by specific cleavages by RNase V1 (cuts based-paired nucleotides only; lane V1). As expected, V1 cuts of the RPE ii. stem are preferentially detected in the strand that is more proximal to the 5′-radiolabel. On the other hand, V1 cuts are only detected in the more distal strand of the longer stem of the double-circle hairpin owing to the fact that the other strand is too close to the 5′ end label (nt 3–7). Since all four G's that are distal to the 5′-radiolabel (namely G_23_, G_31_, G_48_, and G_75_) were predicted to occur in the based paired regions, no cleavages with RNAse T1 (cleaves at 3′ end of single-strand G's) should be detected. The fact that we did reproducibly observe cuts at all four G's (lanes T1) suggests that the 79-mer is metastable, undergoing dynamic unfolding/folding cycles in our sample. This is expected, however, given that the REI process requires the ribosome to smoothly scan through this region before it translates uORF1, stops at its stop codon and primes itself for resumption of scanning. It is understood that under given circumstances a highly stable secondary structure would actually impede swift translational remodeling of this critical region. Indeed, a critical support for the proposed structure identity was provided by the T1 enzyme under denaturing conditions (lane T1 denatur) that showed a substantially stronger T1 cuts compared to the folded sample (lane T1 fold).

### Identification of the fourth REI-promoting element within the 5′ enhancer of uORF1 that acts in synergy with the RPE i. in the a/TIF32-NTD–dependent manner

**Figure 4:**
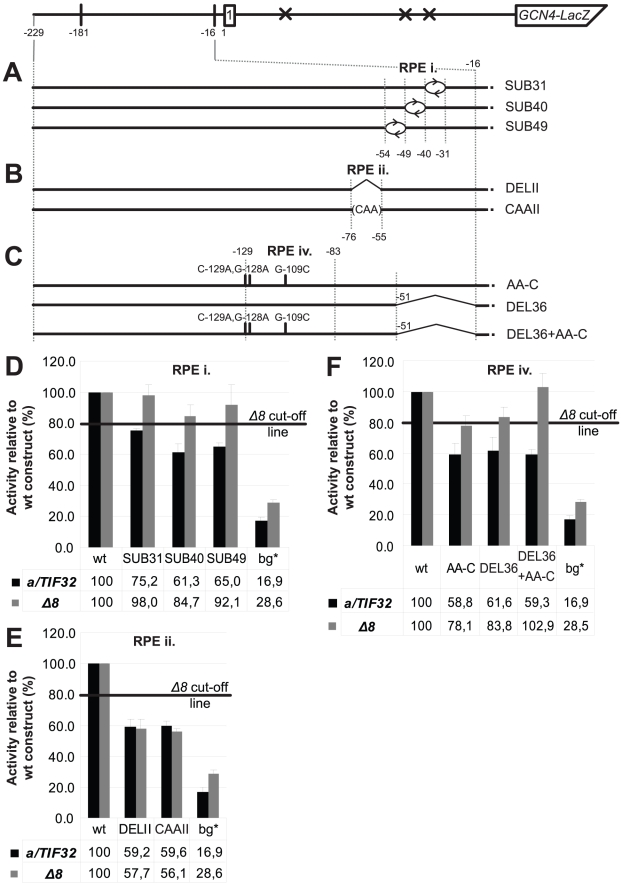
Identification of the forth REI-promoting element within the 5′ enhancer that acts in synergy with the RPE i. in the a/TIF32-NTD–dependent manner. (A–C) Schematics showing the solitary uORF1 *GCN4-lacZ* construct with the battery of substitutions and/or deletions in the RPE i. (A), RPE ii. (B), and RPE iv. (C) that are used in the next three panels, respectively. (D) The RPE i. acts in the a/TIF32-NTD-dependent manner. The YBS47 and YBS53 strains were introduced with the *GCN4-lacZ* substitution constructs described in panel A and Figure 1D, and analyzed as in Figure 2B. wt, construct 1111; bg*, construct 4411. (E) The RPE ii. acts in the a/TIF32-NTD-independent manner. The strains as in panel D were introduced with the *GCN4-lacZ* deletion or substitution constructs described in panel B and analyzed as in Figure 2B. (F) The RPE iv. acts in synergy with the RPE i. in the a/TIF32-NTD-dependent manner. The strains as in panel D were introduced with the *GCN4-lacZ* deletion and/or substitution constructs described in panel C and analyzed as in Figure 2B.

Next we subjected individual RPEs to an in-depth analysis in order to provide additional support for their importance in the REI mechanism of *GCN4*. The RPE i. acts in the a/TIF32-NTD-dependent manner and appears to be unstructured. Hence it is highly likely that the putative direct interaction between the a/TIF32-NTD and the RPE i. is sequence specific. To test that, we divided the RPE i. into three consecutive segments with the first two comprising 9 nt (−31 through −39 in SUB31; and −40 through −48 in SUB40), and the third one being composed of 6 nt (−49 through −54 in SUB49) and ending at the base of the RPE ii stem (Figure 4A and Figure 2C). We then substituted sequences of these segments with complementary nt and tested the resulting constructs for efficiency of *GCN4-lacZ* expression. As shown in Figure 4D, whereas neither of the substitutions significantly affected expression in the *a/tif32-Δ8* cells, SUB31 produced ∼25%, and SUB40 and SUB49 even ∼40% reductions, respectively, in wt cells. Hence the results obtained especially with the latter two substitutions nicely correlate with DEL36 that removes the entire element (Figure 2B and Figure 4F) and suggest that mainly the nature of nt situated at the 5′ end of the RPE i. is critical for its function in REI.

The RPE ii. forms a stem with two bulges and does not seem to be involved in the functional interaction of the 5′ enhancer with the a/TIF32-NTD. We designed two constructs one of which removed all stem-forming nt and the other one replaced them with a stretch of multiple CAA triplets, which minimizes formation of secondary structures [23] (Figure 4B). As predicted, both constructs reduced the *GCN4-lacZ* expression by ∼40% in wt as well as in *a/tif32-Δ8* cells clearly confirming the importance of this element for resumption of scanning after uORF1 in the a/TIF32-NTD-independent fashion. We also swapped both strands of the stem either preserving the sequences of both bulges or replacing them with complementary nt to find out whether the structure or sequence, or both is important. In either case the *GCN4-lacZ* expression went down by consistent ∼40% in both strains (data not shown), suggesting that certainly the sequence is critical for function of this element. The question of the fold importance could not be satisfactorily answered.

As shown in Figure 2B, removal of the RPEs i. and ii. in DEL46 (−16 through −61) produced ∼60% drop in the β-galactosidase activity in wt cells and any of the larger deletions up to −125 nt that we tested did not make it any worse. These findings may indicate that a nucleotide sequence from the 5′ base of the RPE ii. stem (nt −76) upstream (at least up to nt −125) is dispensable for the 5′ enhancer function in REI. Interestingly, however, our computer modeling suggested that a nucleotide stretch spanning nt −129 through −83 folds into the conserved double-circle hairpin (Figure 2C) that, by definition, would be expected to be functionally important. To test that, we employed computer modeling and designed a triple nucleotide substitution (C-129A, G-128A, G-109C) that should completely disrupt base-pairing between nt forming both stems while preserving the length and the rest of the sequence of this rather long segment intact (Figure 4C). As shown in Figure 4F, the resulting AA-C construct indeed reduced the *GCN4-lacZ* expression by ∼40% but only in the wt cells. In principle, it behaved the same as the RPE i.-deletion construct DEL36 indicating that the RPE i. and this hairpin may closely cooperate with each other and also with the NTD of a/TIF32. If true, then combining DEL36 and AA-C mutations (Figure 4C) should be epistatic; and this was exactly observed (Figure 4F). These findings thus identify a fourth REI-promoting element (RPE iv; −129 through −83) within the 5′ enhancer that adopts a conserved higher-order structure and acts in synergy with the RPE i. and the a/TIF32-NTD.

### The RPEs i., ii., and iv. of the 5′ enhancer are critically required for up-regulation of *GCN4* expression under starvation conditions

All experiments described so far were carried out with *GCN4-lacZ* constructs carrying only uORF1 of the four uORFs from the *GCN4* mRNA leader and under non-starvation conditions. To perform an ultimate test of our findings, we examined effects of selected mutations on *GCN4* induction in wt cells treated with 3-aminotriazole (3-AT; an inhibitor of histidine biosynthetic genes that mimics starvation conditions) using a construct containing uORF1 and uORF4 that together suffice for wt regulation of *GCN4* expression (Figure S2). As described in detail in the Text S1, obtained results underpinned the functional importance of all three major 5′ enhancer's RPEs (i., ii., and iv.) in their task to ensure efficient REI on *GCN4* when cells are starved for nutrients such as amino acids.

### The extreme NTD of a/TIF32 contains two distal regions that promote efficient REI in the 5′ enhancer-dependent manner

**Figure 5:**
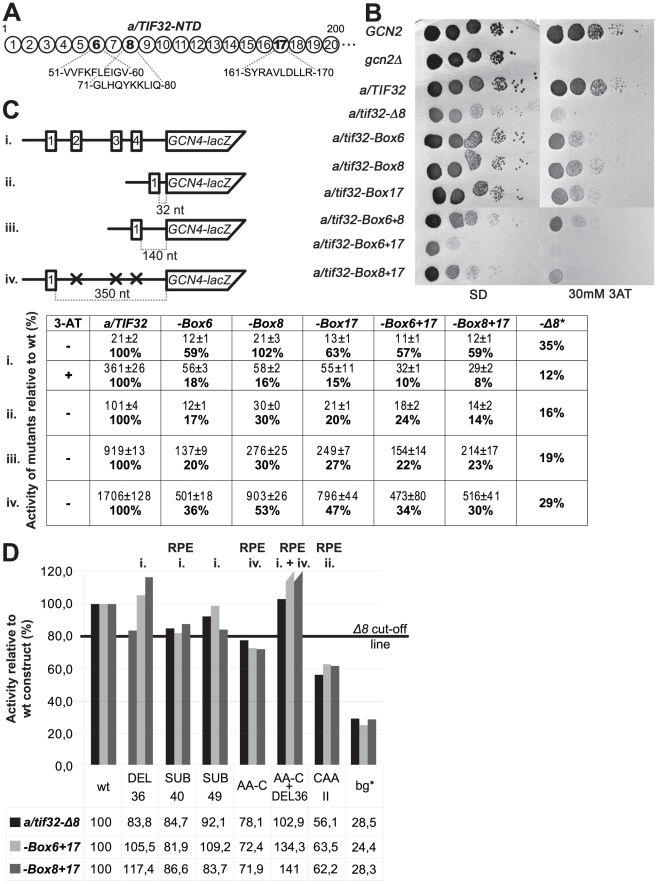
The extreme NTD of a/TIF32 contains two distal regions that promote efficient REI in the 5′ enhancer-dependent manner. (A) Schematic representation of the first 200 amino acid residues of a/TIF32 shown as numbered circles (Boxes 1–20), each of them composed of 10 consecutive residues that were substituted with a stretch of 10 alanines. The sequence of Boxes 6, 8, and 17 is given below the schematic. (B) The *a/tif32-Boxes 6, 8,* and *17* impart a strong Gcn^-^ phenotype. YBS52 (*GCN2 a/tif32Δ*) was transformed with individual YCplac111-based plasmids carrying the indicated a/TIF32 alleles and the resident YCpTIF32-His-U plasmid was evicted on 5-FOA. The resulting strains, together with isogenic strains H2880 (*GCN2 a/TIF32*; row 1) and H2881 (*gcn2Δ a/TIF32*; row 2), were then spotted in five serial 10-fold dilutions on SD (left panel) or SD containing 30 mM 3-AT (right panel) and incubated at 30°C for 3 and 6 days, respectively. (C) The failure of the *a/tif32-NTD-Box* mutations to derepress *GCN4* is caused by a defect in resumption of scanning of post-termination 40S ribosomes on uORF1. Selected strains described in section B were introduced with the *GCN4-lacZ* constructs p180 (i.), pG67 (ii.), pM199 (iii.), and p209 (iv.), respectively, and analyzed as in Figure 2B. To induce the *GCN4-lacZ* expression (section i.), the transformants grown at the minimal media for 2 hrs after dilution were treated with 10 mM 3-AT for 6 hrs (*a/TIF32*) or overnight (*Box* mutants). An asterisk indicates data taken from [10] for comparison purposes. (D) Indicated strains described in section B were introduced with the *GCN4-lacZ* deletion constructs described in Figure 1D and Figure 4A–4C and analyzed as in Figure 2B. The RPEs affected by individual mutations are indicated above the bar diagram. wt, construct 1111; bg*, construct 4411.

The *a/tif32-Δ8* mutation was shown to reduce the REI efficiency by two distinct mechanisms: (i) decreasing retention of eIF3 on elongating ribosomes translating uORF1 by reducing the binding affinity of eIF3 to 40S subunits and (ii) impairing functional interaction of a/TIF32 with the 5′ enhancer of uORF1 [10]. To identify residues in the extreme NTD of a/TIF32 that are responsible for these two roles, and to possibly separate them, we introduced Ala substitutions in consecutive blocks of 10 residues between amino acids 1 and 200 (dubbed Boxes 1 to 20, Figure 5A). None of these mutations was lethal and only Boxes 6 (residues 51–60), 8 (71–80), and 17 (161–170) produced slow-growth (Slg^−^) phenotypes and, most importantly, significant Gcn^−^ phenotypes (Figure 5B) indicating an impairment of the *GCN4* induction. Indeed, our *GCN4-lacZ* reporter assays with the wt *GCN4*-leader confirmed the derepression defect (Figure 5C, construct i). Interestingly, combining Boxes 6+17 and 8+17 but not 6+8 exacerbated both the Slg^−^ and Gcn^−^ phenotypes of the single mutants (Figure 5B and 5C, construct i) suggesting the presence of two functionally partially redundant regions within the a/TIF32-NTD, with the first one represented by Boxes 6 and 8, and the other by Box17. Importantly, in a striking analogy with the *a/tif32-Δ8* mutation [10], all three Boxes as well as their combinations decreased β-galactosidase activities measured from constructs carrying only uORF1 at three different positions relative to *GCN4-lacZ* by a similar number (∼50–80%) (Figure 5C, constructs ii. – vi.) strongly indicating that the failure to induce *GCN4* expression emanates from the inability of 40S subunits to resume scanning after translating uORF1. Remarkably, in contrast to *a/tif32-Δ8,* neither of the Boxes either alone or in pair wise combinations affected the overall eIF3 affinity for 40S subunits *in vivo* (Figure S3A and data not shown). Furthermore, binding of the *in vitro* synthesized a/TIF32-NTD to GST-fused RPS0A was also not affected by these mutations (Figure S3B). Together these findings strongly suggest that the a/tif32-Boxes impact REI specifically by impairing the a/TIF32-NTD interaction with the 5′ enhancer.

To demonstrate directly that the amino acid regions represented by the latter Boxes mediate the REI-promoting interaction between the a/TIF32-NTD and the 5′ enhancer, we analyzed β-galactosidase activities of the selected *GCN4-lacZ* constructs described in Figure 2 and Figure 4 eliminating the key RPEs in the background of the Box6+17 and Box8+17 mutations (Figure 5D and data not shown). Whereas neither DEL36, SUB40 and SUB49 (impairing RPE i.) nor AA-C and DEL36+AA-C (impairing RPE iv. either alone or together with RPE i.) significantly exacerbated deleterious effects of the double-Box mutations on REI efficiency in the mutant cells, CAAII impairing eIF3-independent RPE ii. showed an additive effect when combined with either of the double-Box mutations. These results thus clearly corroborate identification of the two critical 5′ enhancer-dependent regions that together account for the REI-promoting activity of the a/TIF32-NTD independently of its 40S-binding activity.

### The 5′ sequences of the REI-permissive uORF of *YAP1* contain structurally similar features to the RPEs of *GCN4*'s uORF1 and analogously promote efficient REI in concert with the a/TIF32-NTD

Next we asked whether the just described mRNA and protein features required for efficient REI on the *GCN4* mRNA are unique to its uORF1. We took advantage of two genes, *YAP1* and *YAP2*, both encoding stress related transcription factors, the mRNA leaders of which contain short uORF(s) with well described regulatory roles. Whereas the *YAP1*'s uORF permits post-termination 40S ribosomes to efficiently resume scanning for REI on the main ORF (similar to *GCN4*'s uORF1), the uORFs 1 and 2 of *YAP2* act to block ribosomal scanning after their translation by promoting efficient termination followed by rapid mRNA decay [18]. To our knowledge the uORF of *YAP1* is the only short uORF in yeast experimentally proven to promote efficient REI besides *GCN4*'s uORF1; however, in contrast to *GCN4*, the exact link between its REI-mediated translational control mechanism and its stress-protective cellular role(s) is still not fully understood.

**Figure 6:**
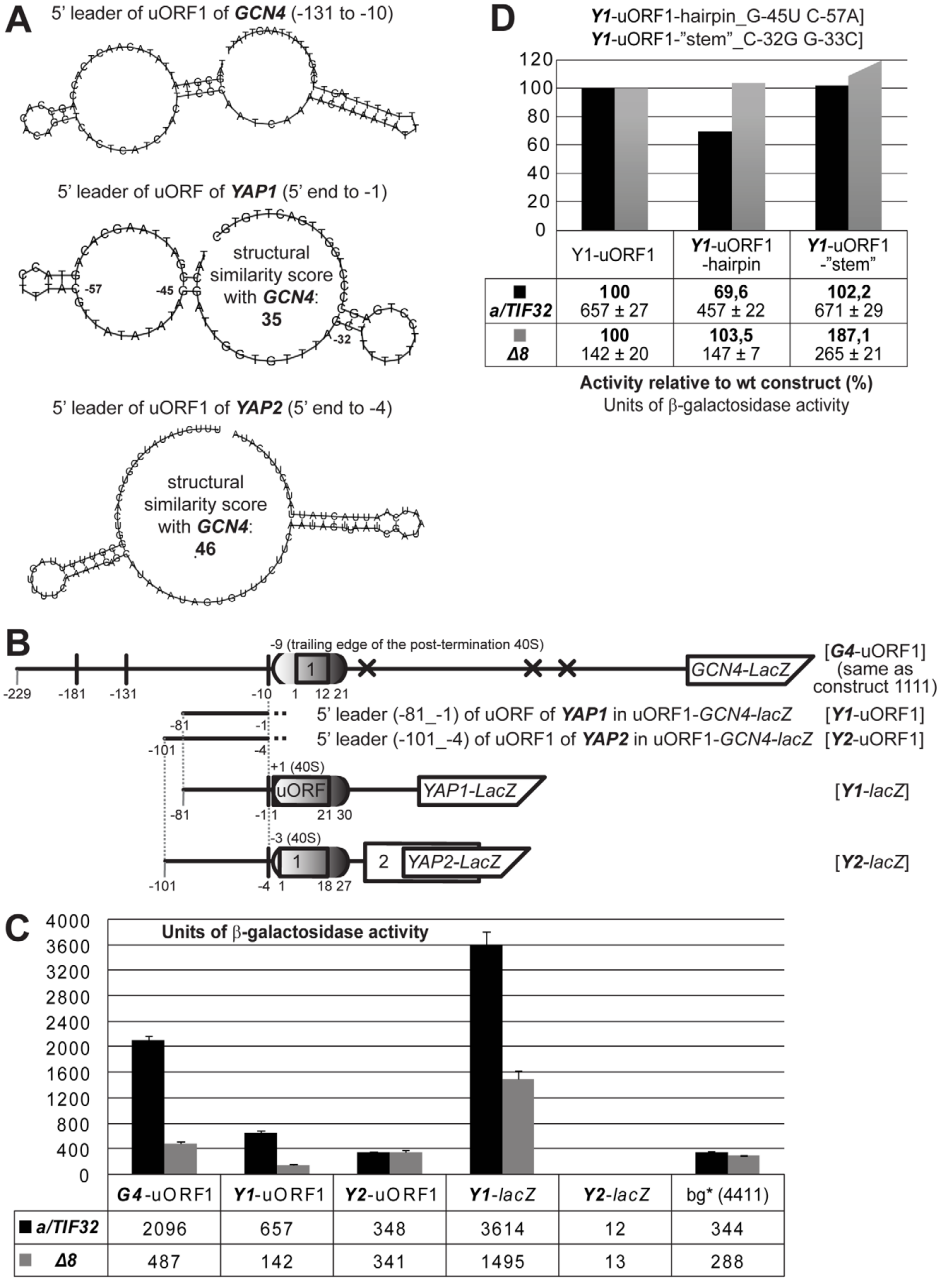
The 5′ sequences of the REI-permissive uORF of *YAP1* contain structurally similar features to the RPEs of *GCN4*'s uORF1 and promote REI in co-operation with the a/TIF32-NTD. (A) *In silico* prediction of secondary structures of the 5′ sequences of uORF1 of *GCN4* (nt −131 through −10), uORF of *YAP1* (nt −81 through −1), and uORF1 of *YAP2* (nt −101 through −4) carried out with the RNA fold software [21]. Pair wise structural similarities of 5′ sequences of *GCN4* with 5′ sequences of *YAP1* and *YAP2* were computed using the RNA distance program [21]. Numbered nucleotides in the *YAP1* sequence indicate mutated positions as illustrated in panel D. (B) Schematic showing the *GCN4-lacZ* construct containing solitary uORF1 (*G4*-uORF1), whose 5′ sequences past the trailing edge of the post-termination 40S ribosome (mRNA exit pore) were replaced by the corresponding 5’ sequences of either uORF of *YAP1* (*Y1*-uORF1) or uORF1 of *YAP2* (*Y2*-uORF1). *YAP1* and *YAP2* constructs where the individual genes were fused with *lacZ* while their 5′ UTRs were kept intact are also shown (*Y1-lacZ* and *Y2-lacZ*, respectively). (C) The YBS47 and YBS53 strains were introduced with the *lacZ* constructs described in panel B and analyzed as in Figure 2B. (D) The YBS47 and YBS53 strains were introduced with two structural mutants in the 5′ sequences of *YAP1* (specified in panel A) inserted into *Y1*-uORF1 and analyzed as in Figure 2B.

We first computationally predicted potential secondary structures of the 5′ sequences of *YAP1*'s uORF (−81 to −1) and of *YAP2*'s uORF1 (−101 to −4) occuring behind the trailing edge (the mRNA exit channel) of the post-termination 40S ribosome, using an analogous folding model as that described for the *GCN4* 5′ sequences above. The predicted secondary structures were compared with that occurring in the corresponding region of *GCN4*′s uORF1 (−131 to −10) (Figure 6A). The structure similarities, computed using the RNA distance program [21], revealed a remarkable resemblance between predicted secondary structures of 5′ sequences of *YAP1*'s uORF and RPEs of *GCN4*'s uORF1; the similarity score reached the value of 35 (compared to 46 for *YAP2* versus *GCN4*; the higher the number, the lower the similarity), which is highly significant considering that the compared sequences are fairly short (∼90 nt). It mainly arises from (i) the occurrence of a double-circle hairpin and (ii) similar lengths of unstructured sequences indicating congruent positioning of the structured elements in the overall folds. It is worth noting that no significant sequence similarities were observed (data not shown) suggesting that these particular structural features might truly play an important role in the REI mechanism.

To examine that, we replaced the entire 5′ leader of uORF1 of *GCN4* excluding the promoter region with the corresponding sequences from both *YAP* genes in our *GCN4-lacZ* construct containing solitary uORF1 (Figure 6B) and measured β-galactosidase activities in wt as well as *a/tif32-Δ8* cells. Whereas the 5′ leader of uORF1 of *YAP2* (in *Y2*-uORF1) showed background levels in both strains, as expected, the 5′ sequences of uORF of *YAP1* (in *Y1*-uORF1) stimulated the *GCN4-lacZ* expression by ∼2-fold over the background in wt cells (Figure 6C). Strikingly, this activity dropped by ∼80% in *a/tif32-Δ8*. The similar reduction was also obtained when we fused the *YAP1* gene with its intact 5′ leader with *lacZ* (in *Y1-lacZ*). In contrast, a *lacZ* fusion with the *YAP2* gene containing its natural 5′ leader (in *Y2-lacZ*) showed no β-galactosidase activity at all in accord with previous observations implicating both uORFs of the *YAP2* mRNA in promoting its rapid degradation [18]. Importantly, point mutations designed to disrupt the conserved double-circle hairpin (in *Y1*-uORF1-hairpin_G-45U C-57A) reduced the *Y1*-uORF1 activity by ∼30% in wt cells and showed the epistatic interaction with *a/tif32-Δ8* (Figure 6D), in good agreement with the data presented in Figure 4F. In contrast, mutations disrupting the predicted non-conserved bulged-stem (in *Y1*-uORF1-“stem”_C-32G G-33C) showed no reduction in either of the strains indicating that it is either not functionally important or not affected by our mutations. Taken together these results clearly demonstrate that the specifically structured 5′ enhancers of REI-permissive uORFs of *GCN4* and *YAP1* are at least partially functionally interchangeable and critically require the NTD of a/TIF32 for their function. Hence a possibility for a common mechanism of translational control operating on short REI-permissive uORFs seems highly likely.

## Discussion

The widespread prevalence of uORFs in mammalian transcriptomes (up to 50%) suggests that REI after translation of a short ORF represents a comprehensive, yet underestimated and grossly unexplored, cis-regulatory function in translational control of gene expression [2]. Indeed, the first examples of aberrant protein expression leading to pathophysiological mechanisms in the etiology of human diseases that are connected to defective uORF-mediated translational control have already been described [2], [24]–[29].

Translational control of yeast *GCN4* transcriptional activator is unarguably the best studied example of the REI mechanism [8]. Particularly intriguing is the fact that only one of its four short uORFs (uORF1) promotes efficient REI thanks to the presence of two specific enhancing sequences (designated here as “enhancers”) flanking its coding region. Importantly, both of these enhancing sequences were previously demonstrated to be transferable [19]; i.e. to function independently of the sequence context of uORF1 indicating that their activity is directly imprinted in their sequence and/or structure. Whereas the mode of action of the 3′ enhancer is not known, the 5′ sequences encompassing a rather long (∼160) stretch of nucleotides were shown to co-operate with the NTD of the a/TIF32 subunit of eIF3 in stabilizing the post-termination 40S subunit on the mRNA [10]. In this study, experiments are described that (i) identify individual stimulatory elements within the 5′ sequences of uORF1, making up the 5′ enhancer, as well as in the NTD of a/TIF32, and (ii) allow us to evaluate their functional importance for the REI mechanism. In addition, our analysis of the 5′ leader of yet another transcriptional activator *YAP1* demonstrates that (iii) the functional interaction between the a/TIF32-NTD and the specifically folded sequences 5′ of a REI-permissive uORF represent a generally applicable requirement for efficient REI at least in yeast.

### Modeling the events that, following termination of translation of uORF1, are required for subsequent resumption of scanning

We first tested the individual contributions of both uORF1's enhancers on efficiency of REI by their individual replacements with the corresponding sequences of the REI-nonpermissive uORF4. Previously, a similar cassette replacement mutagenesis was carried out [19]; however, it did not include the uORF1's 5′ sequences. In accordance with Grant et al. [14], the uORF1's 3′ enhancer alone (Figure 1D; construct 4411) was still capable to allow some REI on *GCN4-lacZ* (∼4-fold higher than the background control in uORF4; construct 4444), albeit the overall REI activity was strongly reduced by ∼80% when compared to wt (construct 1111). On the contrary, the REI activity of the 5′ enhancer alone containing all four RPEs (construct 1144) dropped to the background levels of uORF4. Thus rather than making additive contributions to the uORF1's ability to support a high frequency of REI at *GCN4*, as originally proposed, it seems that the 3′ enhancer acts first and its action is a prerequisite for the subsequent contribution of the 5′ enhancer. We propose the following model of the sequence of events on the uORF1 that follow termination of its translation and that, in the light of our *YAP1* data, could be applicable to short uORFs with the REI-permissive character in general (Figure 1A and 1B).

Upon stop codon recognition, the 3′ enhancer, buried for its most part in the mRNA binding channel, interacts somehow with the ribosome and ensures that the 40S subunit remains attached to the mRNA during the first ribosomal recycling reaction that removes the large ribosomal subunit and is thought to be catalyzed by RLI1/ABCE1 [30]. This alone suffices for a certain level of elevated efficiency of REI. In the meantime, the 5′ enhancer that has gradually emerged from the mRNA exit channel progressively folds into its secondary/tertiary structure and contacts the a/TIF32-NTD, previously shown to interact with RPS0A occurring near the mRNA exit pore [10], [15], to further stabilize the 40S subunit on the *GCN4* mRNA. This second step considerably boosts the efficiency of REI as it prevents recycling of at least 50% of small subunits [14]. Consistent with our model, mammalian eIF3a was shown to interact with mRNA in the 48S PIC in a way extending the mRNA binding channel beyond the exit site [31]. In addition to a/TIF32, the g/TIF35 subunit of eIF3 also promotes this process, however, by an unknown mechanism that does not depend on the 5′ enhancer and awaits a detailed investigation [17]. Interestingly, plant eIF3g together with eIF3h were similarly shown to support efficient REI [32], [33], however, their mechanistic contributions also remain to be explored. Once the mRNA-40S complex is sufficiently stabilized, eIF3 most probably facilitates recruitment of scanning-promoting factors namely eIF1 and eIF1A. These factors were shown to trigger conformational changes of the 40S head region resulting in the open/scanning conducive conformation that is required for linear scanning from the mRNA′s 5′ cap [34]. It is very likely that similar conformational changes are also needed for the mRNA-bound post-termination 40S subunit in order to resume scanning.

How the 3′ enhancer performs its initial task is currently under investigation in our laboratory. Previous work suggested that its AU-rich content (∼60%) rather than a particular sequence could be critical for its function. In fact, it was proposed that the AU-rich sequence would not form strong base-pairing interactions with the 40S subunit and would allow it to promptly resume scanning [13]. However, with the exception of uORF4 (AU-content ∼40%), the sequences corresponding to the 3′ enhancer of other two *GCN4*′s uORFs (2 and 3) have even higher AU-content (∼85% and ∼70%, respectively), yet they do not promote REI as uORF1. Besides, our model posits that the ribosome terminating on uORF1 spends longer than usual time on the termination/recycling steps to allow the 5′ enhancer to fold and interact with the a/TIF32-NTD. Hence we think that a simple enrichment in A and U nt is unlikely to be the key to this puzzle, and it is still possible that the 3′ enhancer contains a less stringent sequential motif that contacts some component of the post-termination complex, presumably 18S rRNA (Figure 1B).

If true, this mechanism would bear a significant resemblance to the termination/reinitiation mechanism that is the best described for the polycistronic mRNA of feline calicivirus [35], [36]. A specific 87-nt element (called TURBS) preceding the overlapping termination/initiation site of two long ORFs 2 and 3 folds into a specific secondary structure that in fact resembles our double-circle hairpin. A part of this structure interacts with a complementary segment of 18S rRNA and also with eIF3 via several subunits including eIF3a and eIF3g to prevent dissociation of the mRNA/eIF3/40S complex in order to allow efficient REI on ORF3. Even though this system operates on long ORFs, its mechanistic likeness with the short uORF-mediated REI does not seem to be accidental from the evolutionary point of view.

### The 5′ enhancer of uORF1 contains four REI-promoting elements, two of which act in synergy in the a/TIF32-NTD–dependent manner

The original data by the Hinnebusch′s group suggested that the ∼160 nt-long 5′ sequences may contain two critical REI-promoting motifs [14]. In agreement, we identified not only two but together four individual elements denoted RPEs that together account for the stimulatory effect of the uORF1's 5′ enhancer on REI. Individual mutations of the unstructured RPE i. and the structured RPE iv., as well as the combination of these mutations were found to be epistatic with the *a/tif32-Δ8* mutant. These results clearly suggest that both elements are needed to contact the a/TIF32-NTD and thus it seems conceivable that they might fold together in a higher-order structure. The fact that the RPE iv. is structurally conserved among various yeasts (JP and LV, unpublished observations) may suggest that the RPE iv. provides a structural basis for the 5′ enhancer–a/TIF32 interaction, whereas the RPE i. lends a specificity to it. Whether it is a direct interaction is currently being explored in our laboratory in the living cells.

In addition to that, our genetic epistasis experiments revealed that the RPEs i. and iv. interact with the a/TIF32-NTD via its two relatively distal REI-promoting regions represented by Boxes 6 and 8, and by Box17, respectively (Figure 5). Importantly, neither of these regions mediates a direct contact of a/TIF32-NTD with RPS0A to facilitate eIF3 binding to 40S ribosomal subunits *in vivo* (Figure S3) clearly suggesting that they promote efficient REI solely in the 5′ enhancer-dependent manner. Interestingly, unlike in the case of RPEs i. and iv., combination of mutations in both of these REI-promoting regions exacerbated the effect of the individual mutations. Hence it seems likely that even though each region may contact the 5′ enhancer individually, their mutual co-operation is required to establish a strong interaction.

Mutations in RPEs ii. and iii. showed additive effects when combined with *a/tif32-Δ8* indicating that the molecular mechanism of their involvement in REI differs from that of RPEs i. and iv. The model structure predicts that the RPE ii. forms a 9 nt-long stem loop whose sequence and less likely also the structure are crucial for its stimulatory activity. At present we can only speculate about the molecular nature of the roles of these two RPEs. They could either contact other eIF3 subunits or other eIFs, or act independently, for example by interacting directly with the ribosomal components.

### Is the critical involvement of the a/TIF32 subunit of eIF3 and the sequences upstream of a short uORF a general requirement for the efficient REI?

Even though there is an increasing number of short uORFs demonstrated to permit efficient REI after their translation [12], [28], [37], [38], perhaps none of them, besides uORF1 of *GCN4* [8], [10], has been studied deeply enough to draw any general conclusions regarding the molecular details of the short uORF-mediated REI mechanism. Until now, this has also applied on the only other well defined REI-permissive uORF in yeast occuring in the mRNA leader of the transcription factor *YAP1* [18]. Here we showed that its 5′ sequences share significant structural similarity predictions with *GCN4*′s uORF1 and, most importantly, stimulate REI on *YAP1* in a strict dependency on the NTD of a/TIF32 (Figure 6). Hence the functional if not direct interaction between the a/TIF32-NTD and the specifically folded sequences upstream of a short REI-permissive uORF represents the first generally applicable requirement of this type of a regulatory mechanism described to date, at least in yeast. Considering the remarkable similarity with the aforementioned termination/reinitiation mechanism utilized by viruses, it is very likely that the analogous principles apply also to uORFs promoting efficient REI in higher eukaryotes. Future work exploring the mechanistic details of some of these uORFs, especially those connected with pathophysiological mechanisms, will certainly tell us more about the evolutionary conservation of this important translational control process.

## Materials and Methods

### Yeast strains, plasmids, RNA structure probing, and other biochemical methods

Lists of strains (Table S1), plasmids (Table S2), and PCR primers (Table S3) used in this study and details of their construction can be found in the Text S1.

Commercially synthesized 79-mer RNA (East Port) was 5′-end-labeled using γ-^32^P-ATP and T4 Polynucleotide Kinase (Fermentas). Free radioactive nucleotides were removed by NucAway Spin Columns (Ambion). The RNA was then subjected to limited digestion with RNase T1 (cleaves after single-stranded G residues) or RNase V1 (cleaves within double-stranded RNA). RNase T1 was used in RNA Sequencing buffer or in RNA Structure buffer to induce denaturing (denatur) or folding-promoting (fold) conditions, respectively. Alkaline hydrolysis of the RNA was used to generate appropriate reference landmarks. (All enzymes, buffers and protocols were provided by Ambion). The digested products were then separated on 10% polyacrylamide (8M urea) sequencing gel in 1xTBE buffer.

β-galactosidase assays were conducted as described previously [13]. GST-pull-down experiments, preparation of whole-cell extracts, sucrose gradient separations and Western blot analysis of gradient fractions were essentially conducted as described in [39], [40].

## Supporting Information

Figure S1 The 5′ sequences of uORF1 contain at least three REI-promoting elements (RPEs), one of which operates in the a/TIF32-NTD-dependent manner. (A) Schematic showing the solitary uORF1 GCN4-lacZ construct with the battery of deletions in the uORF1's 5′UTR defined below and used in panel B. (B) The YBS47 and YBS53 strains were introduced with the GCN4-lacZ deletion constructs described in panel A and analyzed as in Figure 2B. This panel is showing identical data to those presented in Figure 2B except that the obtained values were not expressed relative to the value obtained with the wt uORF1-*GCN4-lacZ* construct. (C) Quantitative RT-PCR of the selected *GCN4-lacZ* transcripts shows no significant changes in their stability. Primers matching the *lacZ* fusion gene were used for quantification. *ADH1* was used as an internal normalization standard. Values obtained for individual mutant constructs in triplicates are expressed relative to the value obtained with the wt *GCN4-lacZ* construct p209. Error bars = SD.
(EPS)

Figure S2 The RPEs i., ii., and iv. of the 5′ enhancer are critically required for up-regulation of GCN4 expression under starvation conditions. (A) Schematic showing the inducible uORF1 – uORF4 GCN4-lacZ construct with the selected substitutions and/or deletions in the color-coded RPEs that are used in panel B. (B) The YBS47 strain was introduced with the GCN4-lacZ deletion and/or substitution constructs described in panel A and analyzed as in Figure 1D. To induce the *GCN4-lacZ* expression, the transformants grown at the minimal media for 2 hrs after dilution were treated with 10 mM 3-AT for 6 hrs. wt^#^, construct 1111 (pM23); bg^#^, construct 4411 (pVM37).
(EPS)

Figure S3 The a/tif32-Box6 and Box17 mutations neither decrease the overall eIF3 affinity for 40S subunits *in vivo* nor reduce binding of the a/TIF32-NTD to the small ribosomal protein RPS0A *in vitro.* (A) Isogenic strains derived from YBS52 *(GCN2 a/tif32Δ)* replacing the resident YCpTIF32-His-U plasmid by YCp-a/TIF32-His-screen, YCp-a/tif32-Box6-His or YCp-a/tif32-Box17-His, respectively, as described in Figure 5B were grown in YPD medium at 30°C to an OD_600_ of ∼1–1.5 and cross-linked with 2% HCHO prior to harvesting. WCEs were sedimented through 7.5 to 30% sucrose gradients, collected fractions were pooled as indicated and subsequently subjected to Western analysis with antibodies against the denoted proteins. An aliquot of each WCE was analyzed in parallel (In, input). The amounts of each factor in the 43S fractions (boxed) obtained from three independent experiments were normalized for the RPS0A level and the ratios of the eIF/40S levels in the mutant to those in the WT were averaged. The means and standard errors are plotted in the histogram. (B) RPS0A fused to GST (lane 3) or GST alone (lane 2) were tested for binding to the ^35^S-labeled a/TIF32-NTD (amino acid residues 1–400) and its mutant derivatives in GST pull down assays. The GST proteins were visualized by Coomassie blue staining (top); radiolabeled proteins by autoradiography (bottom). Lane 1 contains 20% of the input amounts of corresponding *in vitro* translated proteins used in the individual binding reactions.
(TIF)

Table S1 Yeast strains used in this study.
(DOCX)

Table S2 Plasmids used in this study.
(DOCX)

Table S3 Oligonucleotides used in this study.
(DOCX)

Text S1 Supporting Results and Materials and Methods.
(DOC)
