# Supplementary figures and images for: Translation Reinitiation Relies on the Interaction between eIF3a/TIF32 and Progressively Folded cis-Acting mRNA Elements Preceding Short uORFs
Source: PLoS Genet. 2011 Jul 7;7(7):e1002137. doi: 10.1371/journal.pgen.1002137 (PMC3131280; doi:10.1371/journal.pgen.1002137)

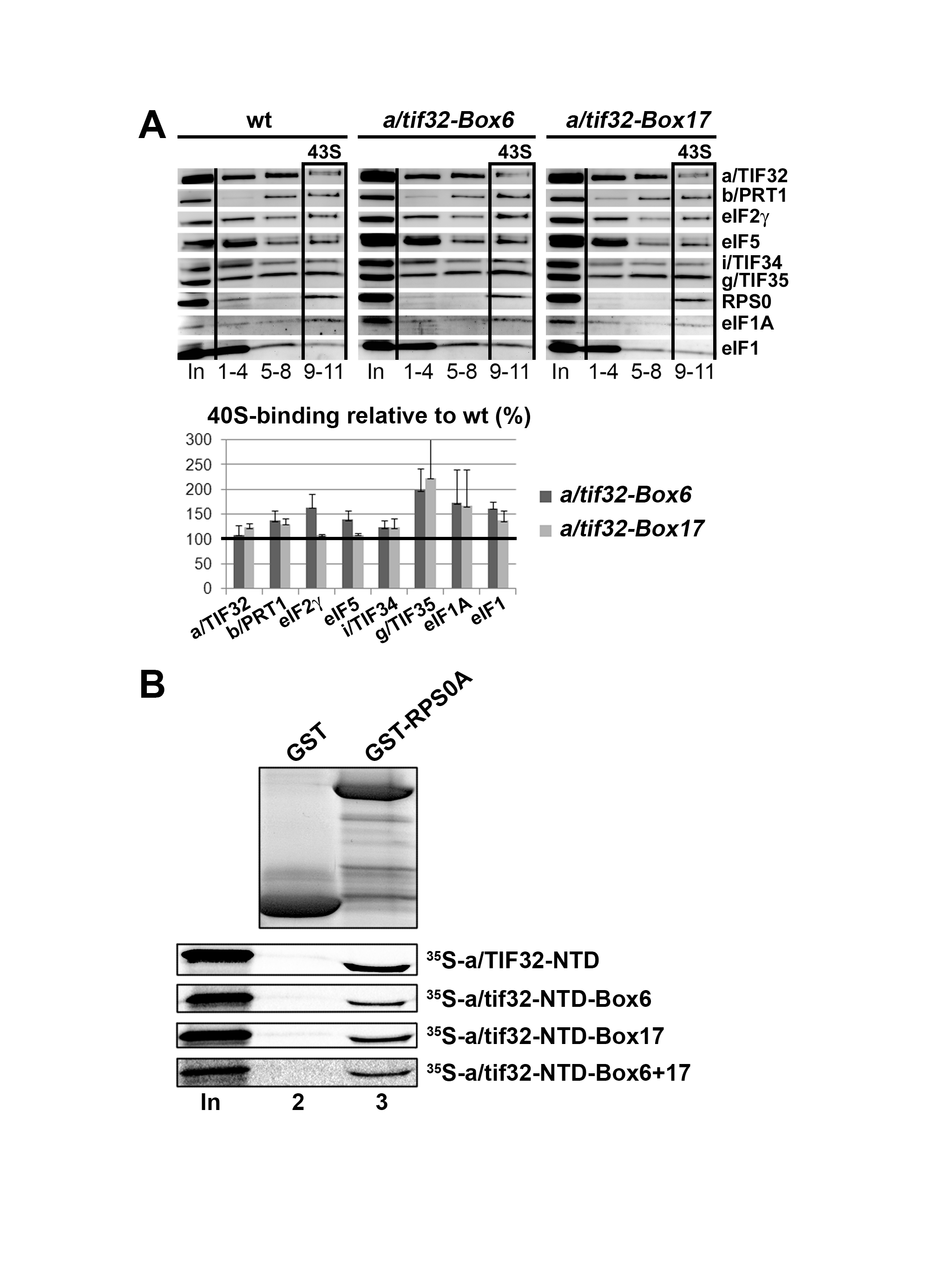

Supplement: Figure S3 — The a/tif32-Box6 and Box17 mutations neither decrease the overall eIF3 affinity for 40S subunits in vivo nor reduce binding of the a/TIF32-NTD to the small ribosomal protein RPS0A in vitro. (A) Isogenic strains derived from YBS52 (GCN2 a/tif32Δ) replacing the resident YCpTIF32-His-U plasmid by YCp-a/TIF32-His-screen, YCp-a/tif32-Box6-His or YCp-a/tif32-Box17-His, respectively, as described in Figure 5B were grown in YPD medium at 30°C to an OD600 of ∼1–1.5 and cross-linked with 2% HCHO prior to harvesting. WCEs were sedimented through 7.5 to 30% sucrose gradients, collected fractions were pooled as indicated and subsequently subjected to Western analysis with antibodies against the denoted proteins. An aliquot of each WCE was analyzed in parallel (In, input). The amounts of each factor in the 43S fractions (boxed) obtained from three independent experiments were normalized for the RPS0A level and the ratios of the eIF/40S levels in the mutant to those in the WT were averaged. The means and standard errors are plotted in the histogram. (B) RPS0A fused to GST (lane 3) or GST alone (lane 2) were tested for binding to the 35S-labeled a/TIF32-NTD (amino acid residues 1–400) and its mutant derivatives in GST pull down assays. The GST proteins were visualized by Coomassie blue staining (top); radiolabeled proteins by autoradiography (bottom). Lane 1 contains 20% of the input amounts of corresponding in vitro translated proteins used in the individual binding reactions. (TIF) [file pgen.1002137.s003.tif]
