# Supplementary material for: Translation Reinitiation Relies on the Interaction between eIF3a/TIF32 and Progressively Folded cis-Acting mRNA Elements Preceding Short uORFs
Source: PLoS Genet. 2011 Jul 7;7(7):e1002137. doi: 10.1371/journal.pgen.1002137 (PMC3131280; doi:10.1371/journal.pgen.1002137)
Supplement: Table S1 — Yeast strains used in this study. (DOCX) [file pgen.1002137.s004.docx]

**Table S1.** Yeast strains used in this study.

| **Strain** | **Genotype** | **Source or reference** |
| --- | --- | --- |
| YBS47^a^ | *MAT*a *leu2-3, -112 ura3-52 trp1Δ gcn2Δ tif32Δ URA3::GCN2 ura3* (pRS-a/TIF32-HIS-L) | [2] |
| YBS53 ^a^ | *MAT*a *leu2-3, -112 ura3-52 trp1Δ gcn2Δ tif32Δ URA3::GCN2 ura3* (pRS-a/tif32-Δ8-HIS-L) | [2] |
| YBS52 ^a^ | *MAT*a *leu2-3, -112 ura3-52 trp1Δ gcn2Δ tif32Δ URA3::GCN2 ura3* (Ycp-a/TIF32-His-U) | This study |
| H2880^a^ | MATa trp1Δ leu2-3,112 ura3-52 | [9] |
| H2881 ^a^ | MATa trp1Δ leu2-3,112 ura3-52 gcn2::hisG | [9] |

^a^ Isogenic strains.
