# Supplementary material for: Translation Reinitiation Relies on the Interaction between eIF3a/TIF32 and Progressively Folded cis-Acting mRNA Elements Preceding Short uORFs
Source: PLoS Genet. 2011 Jul 7;7(7):e1002137. doi: 10.1371/journal.pgen.1002137 (PMC3131280; doi:10.1371/journal.pgen.1002137)
Supplement: Table S2 — Plasmids used in this study. (DOCX) [file pgen.1002137.s005.docx]

**Table S2.** Plasmids used in this study.

| **Plasmid** | **Description** | **Source of reference** |
| --- | --- | --- |
| pM128  (1-1-1-1) | low copy URA3 vector containing uORF1 only at its original position (350 nt from *GCN4-lacZ*), with *Hind*III restriction site upstream of uORF1 | [4] |
| pM114  (1-1-4-4) | low copy URA3 vector containing uORF1 only (with *Hind*III upstream); the coding sequence and 25 nt of its 3' flanking sequence was replaced by the corresponding sequences of uORF4 | [5] |
| pVM35  (4-4-4-4) | low copy URA3 vector containing uORF1 only (with *Hind*III upstream); uORF1’s 5’ flanking sequences (nt -181 to -1), the coding region, and the 3’ flanking sequences (25 nt beyond the stop codon) were replaced by the corresponding sequences of uORF4 | This study |
| pVM36  (4-4-1-1) | low copy URA3 vector containing uORF1 only only (with *Hind*III upstream); ); uORF1’s 5’ flanking sequences (nt -181 to -1) were replaced by the corresponding sequences of uORF4 | This study |
| pVM15  (DEL6) | low copy URA3 vector containing uORF1 only at its original position; the sequences -21 to -16 upstream of uORF1 were deleted | This study |
| pVM14  (DEL16) | low copy URA3 vector containing uORF1 only at its original position; the sequences -31 to -16 upstream of uORF1 were deleted | This study |
| pVM13  (DEL26) | low copy URA3 vector containing uORF1 only at its original position; the sequences -41 to -16 upstream of uORF1 were deleted | This study |
| pVM12  (DEL36) | low copy URA3 vector containing uORF1 only at its original position; the sequences -51 to -16 upstream of uORF1 were deleted | This study |
| pVM11  (DEL46) | low copy URA3 vector containing uORF1 only at its original position; the sequences -61 to -16 upstream of uORF1 were deleted | [2] |
| pVM16  (DELup39) | low copy URA3 vector containing uORF1 only at its original position; the sequences -181 to -143 upstream of uORF1 were deleted | This study |
| pVM18  (DEL56) | low copy URA3 vector containing uORF1 only at its original position; the sequences -71 to -16 upstream of uORF1 were deleted | This study |
| pVM20  (DEL76) | low copy URA3 vector containing uORF1 only at its original position; the sequences -91 to -16 upstream of uORF1 were deleted | This study |
| pVM21  (DEL109) | low copy URA3 vector containing uORF1 only at its original position; the sequences -125 to -16 upstream of uORF1 were deleted | This study |
| pVM45  (SUB31) | low copy URA3 vector containing uORF1 only at its original position; the sequence -40 to -32 upstream of uORF1 were substituted by complementary sequences | This study |
| pVM46  (SUB41) | low copy URA3 vector containing uORF1 only at its original position; the sequences -49 to -41 upstream of uORF1 were substituted by complementary sequences | This study |
| pVM47  (SUB49) | low copy URA3 vector containing uORF1 only at its original position; the sequences -54 to -50 upstream of uORF1 were substituted by complementary sequences | This study |
| pVM26  (DELII) | low copy URA3 vector containing uORF1 only at its original position; the sequences -76 to -55 upstream of uORF1 were deleted | This study |
| pVM27  (CAAII) | low copy URA3 vector containing uORF1 only at its original position; the sequences -76 to -55 upstream of uORF1 were substituted by a stretch of CAA triplets of the identical length to the original sequence | This study |
| pVM31  (AA-C) | low copy URA3 vector containing uORF1 only at its original position; G-129A, C-128A and G-109C substitutions were inserted upstream of uORF1 | This study |
| pVM50  (DEL36+AA-C) | low copy URA3 vector containing uORF1 only at its original position; the uORF1’s mutations DEL36 and AA-C were combined | This study |
| pVM80  (G4-uORF1) | low copy URA3 vector containing uORF1 only at its original position (350 nt from *GCN4-lacZ*) with *Sac*II restriction site upstream of uORF1 | This study |
| pVM91  (Y1-uORF1) | low copy URA3 vector containing uORF1 only at its original position; the 5’ UTR of uORF1 of *GCN4* (-229_-9) was replaced by the corresponding sequence of YAP1 (-81_-9) | This study |
| pVM92  (Y2-uORF1) | low copy URA3 vector containing uORF1 only at its original position; the 5’ UTR of uORF1 of *GCN4* (-229_-9) was replaced by the corresponding sequence of *YAP2* (-101_-9) | This study |
| pVM95  (Y1-lacZ) | low copy URA3 vector containing the *YAP1* gene with its complete 5’ UTR fused with *lacZ* under control of the *GCN4* promoter | This study |
| pVM96  (Y2-lacZ) | low copy URA3 vector containing the *YAP2* gene with its complete 5’ UTR fused with *lacZ* under control of the *GCN4* promoter | This study |
| pVM97  (Y1-uORF1-“stem”_C-32G G-33C) | a derivative of pVM91; C-32G and G-33G substitutions were inserted upstream of uORF1 | This study |
| pVM98  (Y1-uORF1-hairpin_G-45U C-57A) | derivative of pVM91, G-45U and C-57A substitutions were inserted upstream of uORF1 | This study |
| p180  (YCp50–GCN4–lacZ) | low copy URA3 vector containing wild-type *GCN4* leader | [10] |
| pG67 | low copy URA3 vector containing uORF1 only placed 32 nt from *GCN4-lacZ* | [11] |
| pM199 | low copy URA3 vector containing uORF1 only at the position of uORF4 (140 nt from *GCN4-lacZ*) | [11] |
| p209 | low copy URA3 vector containing uORF1 only at its original position (350 nt from *GCN4-lacZ*) | [4] |
| pM23 (supp info)  (wt^#^; 1-1-1-1) | low copy URA3 vector containing uORF1 and uORF4 only at their original positions relative to *GCN4-lacZ* | [5] |
| pVM55 (supp info)  (DEL16) | derivative of pM23; mutation DEL16 introduced upstream of uORF1 | This study |
| pVM54 (supp info)  (DEL36) | derivative of pM23; mutation DEL36 introduced upstream of uORF1 | This study |
| pVM59 (supp info)  (SUB49) | derivative of pM23; mutation SUB49 introduced upstream of uORF1 | This study |
| pVM60 (supp info)  (DELhairpin) | derivative of pM23; deletion of a double-circle hairpin (nt -129 to -83) upstream of uORF1 | This study |
| pVM61(supp info)  (DELII) | derivative of pM23; mutation DELII introduced upstream of uORF1 | This study |
| pVM56 (supp info)  (CAAII) | derivative of pM23; mutation CAAII introduced upstream of uORF1 | This study |
| pVM53 (supp info)  (DEL46) | derivative of pM23; mutation DEL46 introduced upstream of uORF1 | This study |
| pVM52 (supp info)  (DELup39) | derivative of pM23; mutation DELup39 introduced upstream of uORF1 | This study |
| pVM37 (supp info)  (bg^#^; 4-4-1-1) | derivative of pM23; mutation 4-4-1-1 introduced upstream of uORF1 | This study |
| YCplac111 | single-copy cloning vector, LEU2 | [7] |
| pRS-a/TIF32-His | low-copy a/TIF32-His in LEU2 plasmid, from pRS315 | [6] |
| YCp-a/TIF32-His-L | single-copy a/TIF32-His in LEU2 plasmid, from YCplac111 | This study |
| YCp-a/TIF32-His-screen | single-copy a/TIF32-His with BamHI and NdeI sites introduced just in front of the start codon of a/TIF32 in LEU2 plasmid, from YCplac111 | This study |
| YCp-a/tif32-Box6-His | single-copy a/tif32-Box6-His in LEU2 plasmid, from YCplac111 | This study |
| YCp-a/tif32-Box8-His | single-copy a/tif32-Box8-His in LEU2 plasmid, from YCplac111 | This study |
| YCp-a/tif32-Box17-His | single-copy a/tif32-Box17- His in LEU2 plasmid, from YCplac111 | This study |
| YCp-a/tif32-Box6+8-His | single-copy a/tif32-Box6+8- His in LEU2 plasmid, from YCplac111 | This study |
| YCp-a/tif32-Box6+17-His | single-copy a/tif32-Box6+17- His in LEU2 plasmid, from YCplac111 | This study |
| YCp-a/tif32-Box8+17-His | single-copy a/tif32-Box8+17- His in LEU2 plasmid, from YCplac111 | This study |
| pGADT7 (supp info) | cloning vector for GAL4 activation domain fusion followed by T7 promoter,*LEU2* | CLONTECH |
| pGAD-a/TIF32-NTD (supp info) | a/TIF32-NTD cloned under T7 promoter, from pGADT7 | This study |
| pGAD-a/tif32-NTD-Box6 (supp info) | a/tif32-NTD-Box6 cloned under T7 promoter, from pGADT7 | This study |
| pGAD- a/tif32-NTD-Box17 (supp info) | a/tif32-NTD-Box17 cloned under T7 promoter, from pGADT7 | This study |
| pGAD- a/tif32-NTD-Box6+17 (supp info) | a/tif32-NTD-Box6+17 cloned under T7 promoter, from pGADT7 | This study |
| pGEX-RPS0A (supp info) | GST-RPS0A fusion plasmid from pGEX-4T-1 | I.D. and L.V. unpublished data |
