# Supplementary material for: Translation Reinitiation Relies on the Interaction between eIF3a/TIF32 and Progressively Folded cis-Acting mRNA Elements Preceding Short uORFs
Source: PLoS Genet. 2011 Jul 7;7(7):e1002137. doi: 10.1371/journal.pgen.1002137 (PMC3131280; doi:10.1371/journal.pgen.1002137)
Supplement: Table S3 — Oligonucleotides used in this study. (DOCX) [file pgen.1002137.s006.docx]

Table S3. Oligonucleotides used in this study.

| **Oligonucleotide** | **Sequence (5' to 3')** |
| --- | --- |
| AH100-r | ATAATCGGTTTAGCAAGCCATTTTCCGCGGATCTTTAATTTTTTAATACGATA |
| AH101-f | AAAATGGCTTGCTAAACCGATTAT |
| BS126-r | GGCTGATATTCGGACA |
| BS143-r | TAAAGATCTACTTTAAAAACAAAATATAATCG |
| BS144-f | TCGGTCGACGGGGAATAAAG |
| BS155-r | TGTTGTGAGTTTTTGTTTTG |
| BS191-f | CAAAACAAAAACTCACAACATTAAAGTAGATCTATTATTAGAAAATTA |
| BS192-r | AAGAAAGCTTACCTGATAGCAATTGGTAAC |
| LVTBOX17-1r | TTCCCAAGTAAATCTTAACCA |
| LVTBOX17-2 | TGGTTAAGATTTACTTGGGAAgcagctgctgcagcagcagcagcagcagcaAACAACGCTTTGTTGGAAATT |
| LVTBOX6-1r | TGGCTCAACAGTTGAAGGTGT |
| LVTBOX6-2 | ACACCTTCAACTGTTGAGCCAGCAGCCGCTGCAGCAGCAGCAGCAGCAGCAGAACTAAAGAAAGGTAAGCTG |
| LVTBOX8-1r | GTCTTTTAGCAGCTTACCTTT |
| LVTBOX8-2 | AAAGGTAAGCTGCTAAAAGACgcagctgctgcagcagcagcagcagcagcaGGATCTACCGAAGGTTTGGTT |
| LVTIF32-BN | AAACCATACAAACGCCCAGGAAGGATCCATATGGCCCCCCCACCATTCCGT |
| LVTIF32-mut1 | CGCCCAGGAAGGATCCATATG |
| LVTIF32-mut2-r | TGTGTCGACATCAAAGTTAAC |
| LVTIF32-NB-r | ACGGAATGGTGGGGGGGCCATATGGATCCTTCCTGGGCGTTTGTATGGTTT |
| LVTIF32-NsiI-r | ATACTTCAAACAGAAATGCATAGT |
| LVTIF32-PstI | CGCCAAGCTTGCATGCCTGCAGGG |
| Primer D-r | TTTAAAGTTTCATTCCAGCATTAGC |
| SG1-f | GAAGGATCCATATGGCCCCCCCAC |
| SG2-r | CCCCCTCGAGTTAATCAAAGTTAACTTCAATG |
| VM100-f | AAATAAGCATGCCATATAAACATATCTTGAGTATACATAA |
| VM101-r | TATATATCTAGACCTGAGTAGAAGGACTGTTTCC |
| VM102-f | TACCGATTAAGCACAGTACCT |
| VM103-f | TTTCTATATCGGTCCACTGGC |
| VM104-r | CAGGAAAAAAAGCTAAACACCAATACTATATATAACTTAAAGGTACTGTGCTTAATCGGT |
| VM105-f | ATTGGTGTTTAGCTTTTTTTCCTG |
| VM106-r | CCAGGGGCTCAGGAAAAAAACGTAAACACCAATCCTATATATAACGTAAAG |
| VM107-f | TTTTTTTCCTGAGCCCCTGG |
| VM10-r | CAAGCCATTTTTCAATGATCTTTTTAATACGATACTGATAATAACT |
| VM11-r | CAAGCCATTTTTCAATGATCTTTACTGATAATAACTTAATAAACTGAACTA |
| VM12-r | CAAGCCATTTTTCAATGATCTTTACTTAATAAACTGAACTAAAATAAAATA |
| VM13-r | CAAGCCATTTTTCAATGATCTTTCTGAACTAAAATAAAATATTTTGTTTTG |
| VM19-r | CAAGCCATTTTTCAATGATCTTTTTGTTTTGATTGCGAAGTAGATG |
| VM21-r | CAAGCCATTTTTCAATGATCTTTATGAGTGAGCTGTGTGGCTGGTG |
| VM22-r | CAAGCCATTTTTCAATGATCTTTTCGCTAGTGAAACTGATGGGCAAA |
| VM38-f | CAGTTTATTAAGTTATTATCAGTA |
| VM39-r | TACTGATAATAACTTAATAAACTGTTGATTGCGAAGTAGATGAGTGAG |
| VM40-r | TACTGATAATAACTTAATAAACTGATTGTTGTTGTTGTTGTTGTTGTTGATTGCGAAGTAGATGAGTGAG |
| VM45-f | CCACACAGCTCACTCATCTACTTC |
| VM46-r | GAAGTAGATGAGTGAGCTGTGTGGGTGGTGAGTTGTATAATTCTTTAGTGAAACTGATGGGCAAAAAAA |
| VM4-f | TCGGCTCGCTGTCTTACCTTTTAAAATCTTCTACTTCTTGACAGTACTTATCTTCTTATATAATAGATATCGTACGCTGCAGGTCGAC |
| VM70-r | TGATCTTTAATTTTTTAATACGATTGACTAATAAACTTAATAAACTGAACTAAAAT |
| VM71-f | ATCGTATTAAAAAATTAAAGATCA |
| VM72-r | ATTTTTTAATACGATACTGATAATTTGAATTATAACTGAACTAAAATAAAATATTTTG |
| VM73-f | ATTATCAGTATCGTATTAAAAAAT |
| VM74-r | ACGATACTGATAATAACTTAATATTGACAACTAAAATAAAATATTTTGTTTTGA |
| VM75-f | TATTAAGTTATTATCAGTATCGT |
| VM7-f | ATCAGTTTCACTAGCGAATTATAC |
| VM8-r | GTATAATTCGCTAGTGAAACTGATTGTTGTGAGTTTTTGTTTTG |
| VM90-r | ATATATCCGCGGAACCAGGGGCTCAGGAAAAA |
| VM92-r | ATATATCCGCGGAGTATAATAGTAATTGATTATCGATTAAC |
| VM94-r | AGGTACTGTGCTTAATCGGTAAGATAAGTACTGTCAAGAAGTAG |
| VM95-r | GCCAGTGGACCGATATAGAAAAGATAAGTACTGTCAAGAAGTAG |
| VM96-r | TATATAGGATCCCCCTTCTTCTTCGGTTGCT |
| VM97-r | TATATATCTAGAGCCATCATGTAAAGAATACTTTAC |
| VM98-f | AAATAAGCATGCAGCGGGAACTTTATGGAAAAC |
| VM99-r | TATATAGGATCCTTCTGGGCAGTCCTCCTAC |
| VM9-f | AAAGATCATTGAAAAATGGCTTG |
