# Supplementary material for: Translation Reinitiation Relies on the Interaction between eIF3a/TIF32 and Progressively Folded cis-Acting mRNA Elements Preceding Short uORFs
Source: PLoS Genet. 2011 Jul 7;7(7):e1002137. doi: 10.1371/journal.pgen.1002137 (PMC3131280; doi:10.1371/journal.pgen.1002137)
Supplement: Text S1 — Supporting Results and Materials and Methods. (DOC) [file pgen.1002137.s007.doc]

**SUPPORTING INFORMAtion**

## Results

**The RPEs i., ii., and iv. of the 5’ enhancer are critically required for up-regulation of *GCN4* expression under starvation conditions.**

All of our experiments described in Figures 2 and 4 were carried out with *GCN4-lacZ* constructs carrying only the first uORF1 and under non-starvation conditions. Hence to perform an ultimate test of our findings, we examined effects of selected mutations on *GCN4* induction in wt cells treated with 3-AT using a construct containing two terminal uORFs (uORF1 and uORF4) (Figure S2A), which together suffice for nearly wt regulation of the *GCN4* expression [1]. (We did not test these constructs in *a/tif32-Δ8* as the level of *GCN4* induction is very low in these cells [2] and the expected differences would thus be too small to be fairly interpreted.) As shown in Figure S2B, wt uORF1 – uORF4 construct boosts *GCN4* synthesis by >8-fold when the cells are cultivated in the presence of 3-aminotriazole (3-AT), an inhibitor of histidine biosynthetic genes that mimics starvation conditions. Whereas DEL16 reduced the absolute level of *GCN4* expression by insignificant 5%, removal of the a/TIF32-dependent RPE i. in DEL36 resulted in a dramatic drop off (>70%). Strikingly, deletion of the NTD of a/TIF32 in *a/tif32-Δ8* also results in ~70% reduction of the β-galactosidase activity [2] strongly supporting our conclusion that both of these molecules functionally interact with each other. Consistently, a partial substitution of the RPE i. with complementary nt in SUB49 produced ~45% reduction in *GCN4* expression. Deletion of the second a/TIF32-dependent segment in RPE iv. manifested >30% decrease in the β-galactosidase activity (DELhairpin) as well as both the deletion DELII or the CAAII substitution of the RPE ii., whereas removal of the least potent RPE iii. (DEL39up) showed small to none reduction by 8% (Figure S2B). Somewhat surprisingly, combined deletion of both RPE i. and RPE ii. in DEL46 produced as little β-galactosidase activity (~30%) as the deletion of the RPE i. in DEL36 alone, even though DEL46 displayed a more deleterious effect when assayed in a construct lacking uORF4 and under non-starvation conditions (Figure 2B). Nevertheless, the majority of these results nicely demonstrate functional importance of at least RPEs i., ii., and iv. of the 5’ enhancer in its task to promote efficient resumption of scanning of post-termination 40S ribosomes on uORF1 that is critical for REI on *GCN4* when cells are starved for nutrients such as amino acids.

**Materials and Methods**

**Construction of yeast strains and plasmids**

To construct YBS52, YBS47 was transformed with YCp-a/TIF32-His-U [3] and grown in SD medium supplemented with excess of leucine to enable a spontaneous loss of pRS-a/TIF32-His-L. The resulting strain, auxotrophic for leucine, is YBS52.

To construct pVM35 and pVM36 by fusion PCR, the following two pairs of primers were used with pM128 [4] as a template: (i) BS144 / BS155; and (ii) BS191 / BS192. The PCR products thus obtained were used in a 1:1 ratio as templates for a third PCR amplification with primers BS144 and BS192. The resulting PCR products were digested with *Sal*I and *Hind*III and ligated with *Sal*I-*Hind*III cleaved pM113 and pM116, respectively [5].

All plasmids listed below were created with help of fusion PCR using the indicated two pairs of primers and p209 [4] as a template: for pVM15: (i) BS144 / VM10; and (ii) VM9 / BS126; for pVM14: (i) BS144 / VM11 and (ii) VM9 / BS126; for pVM13: (i) BS144 / VM12 and (ii) VM9 / BS126; for pVM12: (i) BS144 / VM13 and (ii) VM9 / BS126; for pVM16: (i) BS144 / VM8 and (ii) VM7 / BS126; for pVM18: (i) BS144 / VM19 and (ii) VM9 / BS126; for pVM20: (i) BS144 / VM21 and (ii) VM9 / BS126; for pVM21: (i) BS144 / VM22 and (ii) VM9 / BS126; for pVM26: (i) BS144 / VM39 and (ii) VM38 / BS126; for pVM27: (i) BS144 / VM40 and (ii) VM38 / BS126; for pVM31: (i) BS144 / VM46 and (ii) VM45 / BS126; pVM45: (i) BS144 / VM70 and (ii) VM71 / BS126; for pVM46: (i) BS144 / VM72 and (ii) VM73 / BS126; for pVM47: (i) BS144 / VM74 and (ii) VM75 / BS126; and for pVM80: (i) BS144 / AH100 and (ii) AH101 / BS126. The PCR products thus obtained were used in a 1:1 ratio as templates for a third PCR amplification with primers BS144 and BS126. The resulting PCR products were digested with *Sal*I and *Bst*EII and ligated with *Sal*I-*Bst*EII cleaved p209. pVM50 was made essentially as described for all the above plasmids except that pVM12 was used as a template with the folowing pairs of primers: (i) BS144 / VM46 and (ii) VM45 / BS126.

To insert the selected 5‘ enhancer mutations into pM23 (Figure S2) containing uORF1 and uORF4 in front of the *GCN4-lacZ* gene, PCR products were obtained from the corresponding plasmids using primers BS144 and BS143, and digested with *Sal*I and *Bgl*II. The resulting fragments were ligated with *Sal*I-*Bgl*II cleaved pM23.

Plasmids pVM91 and pVM92 were produced by fusion PCR with the following pairs of primers and indicated DNA templates. In the case of pVM91 (i) BS144 / VM94 (with p209 as a template) and (ii) VM102 / VM90 (with genomic DNA as a template) were used; for pVM92 we used (i) BS144 / VM95 (with p209 as a template) and (ii) VM103 / VM92 (with genomic DNA as a template) . The PCR products thus obtained were used in a 1:1 ratio as templates for a third PCR amplification with primers BS144 and VM90 or VM92, respectively. The resulting PCR products were digested with *Sal*I and *Sac*II and ligated with *Sal*I-*Sac*II cleaved pVM80.

pVM95 was constructed in two steps. The *Sph*I-*Xba*I fragment from YCp50-GCN4-lacZ-AH (A. Herrmannová and L. Valášek, to be published elsewhere) was replaced with the *Sph*I-*Xba*I digested PCR product created with primers VM98 and VM97 using genomic DNA as a template producing p180-GCN4-Y1-3’UTR. In the second step, fusion PCR was performed with the following pairs of primers and indicated DNA templates: (i) BS144 / VM94 (with p209 as a template) and (ii) VM102 / VM96 (with genomic DNA as a template). The PCR products thus obtained were used in a 1:1 ratio as templates for a third PCR amplification with primers BS144 and VM96. The resulting PCR product was digested with *Sal*I and *Bam*HI and introduced into *Sal*I-*Bam*HI digested p180-GCN4-Y1-3’UTR producing pVM95.

pVM96 was created similarly only using different pair of primers. In the first cloning step VM100 and VM101 were used to create p180-GCN4-Y2-3’UTR. The fusion PCR then employed the following primer pairs: (i) BS144 / VM95 and (ii) VM103 / VM99 for the first round of fusion PCR reactions; and BS144 / VM99 for the final PCR reaction to produce pVM96.

Plasmids pVM97 and pVM98 are mutant derivatives of pVM91. Corresponding mutations were introduced into pVM91 by fusion PCR using pVM91 as a template and the following pairs of primers: (i) BS144 / VM106 and (ii) VM107 / BS126 for pVM97; and (i) BS144 / VM104 and (ii) VM105 / BS126 for pVM98. Primers BS144 and BS126 were then used in the third reaction with a 1:1 ratio of the corresponding PCR products from the first and second reactions as templates. The resulting PCR products was digested with *Sal*I and *Bst*EII and inserted into *Sal*I-*Bst*EII-cut pVM91 to produce pVM97 and pVM98, respectively.

YCp-a/TIF32-His-screen that was used as a template for the Clustered-Alanine Mutagenesis (CAM) of the a/TIF32-NTD was constructed in two steps. First, the *Pst*I-*Sac*I fragment carrying wild-type *a/TIF32-His* from pRS-a/TIF32-His [6] was inserted into *Pst*I-*Sac*I cut YCplac111 [7] to produce YCp-a/TIF32-His-L. YCp-a/TIF32-His-screen was subsequently generated by fusion PCR, introducing *Bam*HI and *Nde*I sites just in front of the AUG start codon of *a/TIF32*, using pRS-a/TIF32-His-L as template and primers LVTIF32-PstI and LVTIF32-NB-r in the first PCR reaction, and primers LVTIF32-BN and LVTIF32-NsiI-r in the second reaction. Primers LVTIF32-PstI and LVTIF32-NsiI-r were used in the third reaction with a 1:1 ratio of PCR products from the first and second reactions as template. The resulting PCR product was digested with *Pst*I and *Nsi*I and inserted into YCp-a/TIF32-His-L digested with the same enzymes.

Derivatives of YCp-a/TIF32-His-screen with mutations *Box1* to *Box20* were generated by fusion PCR. For example, *Box8* was generated using YCp-a/TIF32-His-screen as template and primers LVTIF32-mut1 and LVTBOX8-1r in the first PCR reaction, and primers LVTBOX8-2 and LVTIF32-mut2-r in the second reaction. Primers LVTIF32-mut1 and LVTIF32-mut2-r were used in the third reaction with a 1:1 ratio of PCR products from the first and second reactions as template. The resulting PCR product was digested with *Bam*HI and *Hpa*I and inserted into YCp-a/TIF32-His-L digested with the same enzymes. Plasmids YCp-a/tif32-Box1-His to YCp-a/tif32-Box20-His were generated in a similar fashion using the appropriate primers (Table S3 shows primers only for two more Boxes (6 and 17) that were used in this study).

In analogy with the latter, YCp-a/tif32-Box6+8-His, YCp-a/tif32-Box6+17-His, and YCp-a/tif32-Box8+17-His were constructed by the combination of the following set of primers using YCp-a/tif32-Box6-His and YCp-a/tif32-Box8-His as templates, respectively (LVTIF32-mut1+ LVTBOX8-1r and TIF32-mut2-r + LVTBOX8-2 for *Box6+8*; LVTIF32-mut1+ LVTBOX17-1r and TIF32-mut2-r + LVTBOX17-2 for *Box6+17*; LVTIF32-mut1+ LVTBOX17-1r and TIF32-mut2-r + LVTBOX17-2 for *Box8+17*).

Plasmids pGAD-a/TIF32-NTD, pGAD-a/tif32-NTD-Box6, pGAD-a/tif32-NTD-Box17 and pGAD-a/tif32-NTD-Box6+17 were constructed with help of PCR using the same pair of primers (SG1-f and SG2-r) and YCp-a/TIF32-His-screen, YCp-a/tif32-Box6-His, YCp-a/tif32-Box17-His and YCp-a/tif32-Box6+17-His as templates, respectively. Obtained PCR products were digested with *Nde*I and *Xho*I and ligated into pGADT7 which was linearized using the same restriction enzymes.

**RNA Isolation and Quantitative Real-Time RT-PCR (qPCR)**

Total RNA was isolated from the exponentially grown cells by extraction with hot acidic phenol according to [8]. Expression of specific mRNA was analyzed by qPCR as follows; 1 µg of total RNA was reverse transcribed using RevertAid M-MuLV Reverse transcriptase (NEB) and random hexanucleotides (VBC). Reverse transcriptase was omitted in control (-RT) samples. The resulting cDNA was diluted 5-times with water and a 3 µl aliquot was used as a template for qPCR. qPCR was performed on the Mx3000P (Stratagene) machine using Maxima SYBR Green qPCR Master Mix (Fermentas). *ADH1* was used as an internal housekeeping gene control. Values of crossing points (CPs) were evaluated and corrected according to PCR efficiency for each reaction. The statistical significance of relative expression changes of mRNA levels normalized to a housekeeping gene was analyzed by the pair-wise fixed reallocation randomization test using the REST 2008 software.

**References**

1. Hinnebusch AG (2005) Translational regulation of GCN4 and the general amino acid control of yeast. Annu Rev Microbiol 59: 407-450.

2. Szamecz B, Rutkai E, Cuchalova L, Munzarova V, Herrmannova A, et al. (2008) eIF3a cooperates with sequences 5' of uORF1 to promote resumption of scanning by post-termination ribosomes for reinitiation on GCN4 mRNA. Genes Dev 22: 2414-2425.

3. Valášek L, Mathew A, Shin BS, Nielsen KH, Szamecz B, et al. (2003) The Yeast eIF3 Subunits TIF32/a and NIP1/c and eIF5 Make Critical Connections with the 40S Ribosome in vivo. Genes Dev 17: 786-799.

4. Grant CM, Hinnebusch AG (1994) Effect of sequence context at stop codons on efficiency of reinitiation in *GCN4* translational control. Mol Cell Biol 14: 606-618.

5. Miller PF, Hinnebusch AG (1989) Sequences that surround the stop codons of upstream open reading frames in *GCN4* mRNA determine their distinct functions in translational control. Genes and Development 3: 1217-1225.

6. Valášek L, Nielsen KH, Hinnebusch AG (2002) Direct eIF2-eIF3 contact in the multifactor complex is important for translation initiation in vivo. EMBO J 21: 5886-5898.

7. Gietz RD, Sugino A (1988) New yeast-Escherichia coli shuttle vectors constructed with in vitro mutagenized yeast genes lacking six-base pair restriction sites. Gene 74: 527-534.

8. Collart MA, Oliviero S (2001) Preparation of Yeast RNA: John Wiley & Sons, Inc.

9. Nielsen KH, Szamecz B, Valasek LJ, A., Shin BS, Hinnebusch AG (2004) Functions of eIF3 downstream of 48S assembly impact AUG recognition and GCN4 translational control. EMBO J 23: 1166-1177.

10. Mueller PP, Hinnebusch AG (1986) Multiple upstream AUG codons mediate translational control of *GCN4*. Cell 45: 201-207.

11. Grant CM, Miller PF, Hinnebusch AG (1994) Requirements for intercistronic distance and level of eIF-2 activity in reinitiation on GCN4 mRNA varies with the downstream cistron. Mol Cell Biol 14: 2616-2628.
